# Photo- and Radiation-Induced One-Electron Oxidation of Methionine in Various Structural Environments Studied by Time-Resolved Techniques

**DOI:** 10.3390/molecules27031028

**Published:** 2022-02-02

**Authors:** Bronislaw Marciniak, Krzysztof Bobrowski

**Affiliations:** 1 Center for Advanced Technology, and Faculty of Chemistry, Adam Mickiewicz University, Uniwersytetu Poznanskiego 10, 61-712 Poznan, Poland; 2 Institute of Nuclear Chemistry and Technology, Dorodna 16, 03-195 Warsaw, Poland

**Keywords:** methionine, oxidation, neighboring group effect, hydroxyl radical, triplet state of carboxybenzophenone, one-electron oxidants, pulse radiolysis, laser flash photolysis, peptides, proteins

## Abstract

Oxidation of methionine (Met) is an important reaction that plays a key role in protein modifications during oxidative stress and aging. The first steps of Met oxidation involve the creation of very reactive and short-lived transients. Application of complementary time-resolved radiation and photochemical techniques (pulse radiolysis and laser flash photolysis together with time-resolved CIDNP and ESR techniques) allowed comparing in detail the one-electron oxidation mechanisms initiated either by ^●^OH radicals and other one-electron oxidants or the excited triplet state of the sensitizers e.g., 4-,3-carboxybenzophenones. The main purpose of this review is to present various factors that influence the character of the forming intermediates. They are divided into two parts: those inextricably related to the structures of molecules containing Met and those related to external factors. The former include (i) the protection of terminal amine and carboxyl groups, (ii) the location of Met in the peptide molecule, (iii) the character of neighboring amino acid other than Met, (iv) the character of the peptide chain (open vs cyclic), (v) the number of Met residues in peptide and protein, and (vi) the optical isomerism of Met residues. External factors include the type of the oxidant, pH, and concentration of Met-containing compounds in the reaction environment. Particular attention is given to the neighboring group participation, which is an essential parameter controlling one-electron oxidation of Met. Mechanistic aspects of oxidation processes by various one-electron oxidants in various structural and pH environments are summarized and discussed. The importance of these studies for understanding oxidation of Met in real biological systems is also addressed.

## 1. Introduction

Methionine (Met) is an important sulfur-containing amino acid often playing a protective role in a protein oxidation due to the fact that the thioether group is easily oxidized by many reactive species [1,2]. These processes play a key role in protein oxidation during oxidative stress and biological aging as certain conditions promote the conversion of sulfur radical cations into sulfoxide [3,4]. The first steps of oxidation mechanisms leading to proteome modifications are extremely fast, i.e., they take place within sub-microsecond and microsecond time domains [5]. They involve creation of the very reactive transients such as radicals and radical cations, which are responsible for the subsequent protein damage. For these reasons, it is important to characterize spectrally and kinetically these very first steps. Radiolysis of water and photo-excitation of benzophenone carboxyl derivatives in aqueous solutions provide very convenient source of hydroxyl radicals HO^●^ and excited triplet states (CB*), respectively, which were very useful for studying one-electron oxidation reactions of Met [5,6]. The transient nature of free radicals requires specific time-resolved methods. Therefore, these reactions were followed either in radiation- or photochemical studies by means of pulse radiolysis [7,8,9,10,11,12,13,14], laser flash photolysis [15,16,17,18,19,20,21,22], CIDNP [23,24,25], and ESR techniques [26]. They were the subject of several review articles [6,27,28,29,30], and also chapters in books [31,32,33,34,35], presenting free radical chemistry in amino acids, peptides, and proteins in a more general manner. 

**Figure 1:**
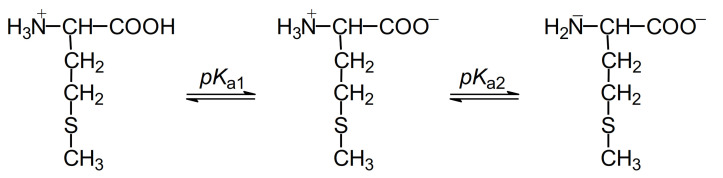
Structures of methionine in aqueous solutions at pH range 1–14.

A wealth of knowledge has been accumulated concerning mechanistic understanding of these processes induced by one-electron oxidation of methionine in various structural environments. To analyze the oxidation mechanism of methionine, one has to take into account that methionine can exist in cationic, zwitterionic, or anionic forms in aqueous solutions depending on the pH range with the respective p*K*_a_ values: (COOH/COO^−^) = 2.3 and (^+^NH_3_/NH_2_) = 9.2 (Figure 1).

**Figure 2:**
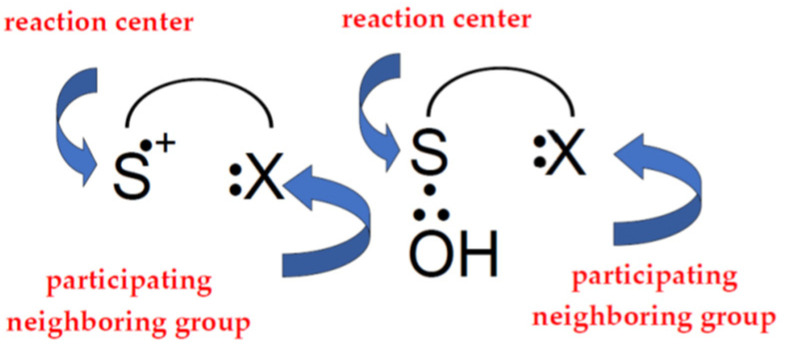
Simplified scheme showing neighboring group participation during oxidation of thioether group of methionine.

Neighboring group participation is also an important concept for understanding how the one-electron oxidation of methionine is controlled. This is the case when a neighboring group stabilizes a transition state or intermediate by becoming bonded to the reaction center as a result of “through space” interaction [36]. These neighboring groups can provide a lone pair of electrons which can be shared with the monomeric sulfur cation center, >S^●+^, forming a three-electron-bonded species which effectively stabilizes the radical cation. The same applies to the ^●^OH-induced oxidation of methionine where the ^●^OH radicals directly attack the sulfur, forming three-electron-bonded adduct (>S∴OH), and its subsequent reactions are strongly influenced by the presence of neighboring groups (Figure 2).

Neighboring group participation in the reactions of two intermediates, monomeric sulfur radical cations (MetS^●+^), and OH-adducts to the sulfur atom (MetS∴OH) during Met oxidation is of great importance, particularly within peptides and proteins. This is due to the presence of a manifold of possible participating functionalities such as carboxyl, amine, hydroxyl, amide, and thioether groups [37]. For a long time it was believed that the intramolecular two-centered, three electron (2c-3e) bonds between the oxidized sulfur atom in Met and the lone electron pair on the nitrogen atom in N-terminal amino group and/or the oxygen atoms in the C-terminal carboxyl group are responsible for stabilization of MetS^●+^). Such stabilization leads to five-membered intramolecularly S∴N-bonded species and/or six-membered intramolecularly S∴O-bonded species. Subsequent studies showed that heteroatoms present in the peptide bond can be also involved in the formation of similar 2c-3e bonds with the oxidized sulfur atom. The general description and spectral characterization of two-centered, three electrons (2c-3e) species was summarized and extensively discussed in numerous papers [38,39,40], book chapters [41,42], and references therein.

The main purpose of this review is to present various factors that might influence the character of intermediates forming during one-electron oxidation of methionine. They can be divided into two parts: those inextricably related to the structure of molecules containing methionine, and those related to the external factors. The former include: (i) protection of terminal amine and carboxyl groups, (ii) location of methionine in the peptide molecule (N/C-terminal and internal), (iii) character of the neighboring amino acid other than methionine, (iv) character of the peptide chain (open vs. cyclic), (v) number of methionine residues in the peptide molecule, and (vi) optical isomerism of methionine residues. In turn, external factors include: (i) type of radiation that induces the oxidation (light vs. high-energy radiation), (ii) type of the oxidant (^●^OH vs one-electron oxidant), and (iii) pH and concentration of Met-containing molecules in the reaction environment. 

Application of complementary radiation and photochemical techniques allowed investigators to compare in detail the one-electron oxidation mechanisms initiated either by ^●^OH radicals and other one-electron oxidants or the excited triplet state of the sensitizer (CB*). The complete and detailed description of the primary steps of the oxidation mechanisms of Met residues located in the interior of long oligopeptides and proteins is a significant and original contribution in understanding oxidation reactions in real biological systems which might be of great help in imagining new strategies in the struggle against “uncontrolled oxidative stress”. The results of these studies and conclusions drawn from them are summarized in this review.

## 2. Methionine and Methionine Derivatives

### 2.1. Radiation-Induced Oxidation

#### 2.1.1. Methionine

In the 1980s, the reactions of ^●^OH radicals and some selected one-electron oxidants with Met were extensively studied by Asmus’ group in order to characterize spectral and kinetic properties of sulfur- and carbon-centered radicals which can be potentially formed [7,43,44,45,46]. Mechanistic studies of the radically induced oxidation of Met revealed rather complex reaction schemes which depend mainly on three parameters, the nature of the oxidant, pH of the solution, and concentration of Met. Although ^●^OH radicals exhibit strong oxidation properties (the standard reduction potentials of the HO^●^/HO^−^ and HO^●^, H^+^/H_2_O redox couples are equal to E^0^ = +1.90 V and +2.72 V vs. NHE, respectively) [47], their oxidation action is not a straightforward one-electron transfer process. Owing to their high electrophilicity the HO^●^ radicals prefer to add to the sulfur atom which is a reaction center of high electron density. 

**Scheme 1:**
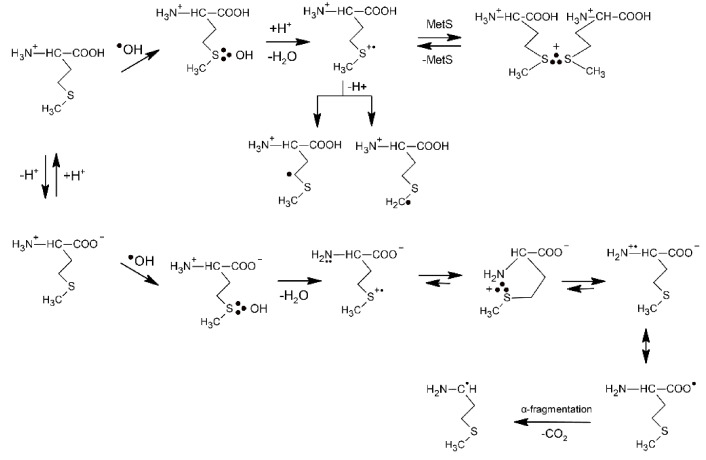
The reactions mechanism for ^●^OH-induced oxidation of methionine (based on [7,46]).

The underlying mechanism of the ^●^OH-induced oxidation of Met is based on an addition of the electrophilic ^●^OH radical to the sulfur atom forming MetS∴OH as the initial step. This reaction occurs with an absolute rate constant *k* = 2.3 × 10^10^ M^−1^ s^−1^ (vide Table 1), irrespective of pH, and is practically only controlled by diffusion of the reactants. The consecutive steps depend further on pH. In strong acid solutions (pH ≤ 3) MetS∴OH will react with external protons yielding MetS^●+^. At a low concentration of Met, MetS^●+^ undergoes deprotonation that leads to α-(alkylthio)alkyl radicals (*k* = 2.4 × 10^5^ s^−1^) (in the following referred to as α-S1 and α-S2 with estimated p*K*_a_(MetS^●^+/Met(−^●^CHSCH_3_) = −6 for deprotonation in the γ-position and p*K*_a_(MetS^●^+/Met(^●^CH_2_SCH_2_−) = −2 for deprotonation in the ε-position [48]. At high concentration of Met, MetS^●+^ can be stabilized via intermolecular equilibration with a second unattacked Met molecule forming a dimeric sulfur radical cation with an intermolecular 2c-3e S∴S bond ((MetS∴SMet)^+^). This dimeric radical cation is characterized by a strong absorption spectrum with λ_max_ = 480 nm. In principle, in acidic conditions Met behaves like an ordinary thioether. At pH ≥ 3, where the carboxyl group is deprotonated, the oxidative mechanism is changed and involves an intramolecular process where protons for dihydroxylation of MetS∴OH are provided by the ^+^NH_3_ group. The latter process leads to formation of an intramolecularly S∴N-bonded intermediate (in the following referred to as S∴N-bonded radical cation, Met(S∴N)^+^) which is assisted by a suitable five membered ring steric arrangement. The Met(S∴N)^+^) is a very-short lived intermediate (τ_1/2_ = 220 ns) and exhibits a transient absorption spectrum with λ_max_ ≈ 390–400 nm [46]. The consecutive reactions include opening of the S∴N-bond to the N-centered radical cation H_2_N^●+^-CHR-COO^−^, establishment of the mesomeric form, H_2_N-CHR-COO^●^ and CO_2_ cleavage of the latter with the formation of 3-methylthiopropylamino radical (in the following referred to as an α-amino radical, α-N). The most important consequences of the mechanism change are decarboxylation of the Met molecule and formation of α-N radicals with strong reductive properties. Formation of the latter radical changes the redox properties of the system from oxidizing to reducing. The general reaction mechanism describing the primary and secondary reactions of radiationally induced ^●^OH radicals in aqueous solutions is presented in Scheme 1.

**Scheme 2:**
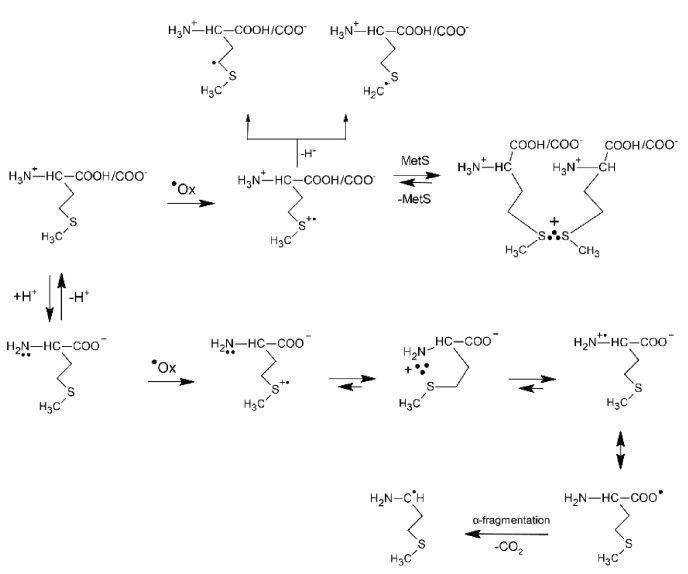
The general reactions mechanism for ^●^Ox−induced oxidation of methionine in aqueous solutions where ^●^Ox = CCl_3_O_2_^●^, CF_3_CHClO_2_^●^, CO_3_^●−^, Tl^2+^, Ag^2+^ (based on [43,45]).

A significantly different picture with respect to pH is observed in the oxidation of Met by the CCl_3_O_2_^●^ radicals (a moderately good one-electron oxidant) [45]. Its reduction potential depends on pH: E^0^(CCl_3_OO^●^, H^+^/CCl_3_OOH) = 1.60 V vs. NHE and E^0^(CCl_3_OO^●^/CCl_3_OO^−^) = 1.15 vs. NHE [49]. The reaction of Met with CCl_3_OO^●^ radicals proceeds with k = 2.9 × 10^7^ M^−1^s^−1^ (vide Table 1). The normalized plots for the formation of the (MetS∴SMet)^+^ and Met(S∴N)^+^ resulting from the oxidation of Met by ^●^OH and CCl_3_O_2_^●^ and based on their absorptions at the respective λ_max_ = 480 and 390 nm clearly indicate that in contrast to the system containing ^●^OH, the dimeric (MetS∴SMet)^+^ radical cations are stabilized over the entire acid and neutral pH range before they are replaced by Met(S∴N)^+^ radical cations. An exclusive formation of the Met(S∴N)^+^ intermediate occurs at pH > 9 in contrast to the reaction of ^●^OH where the Met(S∴N)^+^ intermediate is almost exclusively formed at pH > 4. The apparent change in reaction mechanism occurs in slightly more basic solutions, i.e., near the pK_a2_ of the amino group in Met (vide Figure 1). The formation of Met(S∴N)^+^ in the system containing CCl_3_O_2_^●^ requires the availability of the free electron pair of the unprotonated amino function and therefore this species is only formed beyond and near the pK_a2_ [45]. The oxidation mechanism of Met by CCl_3_O_2_^●^ and also by other one-electron oxidants such as CF_3_CHClO_2_^●^ [45], CO_3_^●−^ [50], Tl^2+^ [43], and Ag^2+^ [43] is exemplified in Scheme 2. Their respective rate constants with Met are listed in Table 1. The parameter which essentially controls whether (MetS∴SMet)^+^ or Met(S∴N)^+^ is formed is the equilibrium constant *K*_a2_ (see Figure 1). However, the kinetics of associated processes have to be also taken into account. 

Although Cl_2_^●−^ radicals exhibit strong oxidation properties (the standard reduction potential of the Cl_2_^●−^/2Cl^−^ redox couple is equal to E^0^ = +2.13 V vs. NHE [47]), their oxidation action is not an adduct mediated one-electron transfer process (an inner-sphere electron transfer) such as in the case of ^●^OH radicals. Moreover, this reaction could be studied only in very acid solutions owing to the complex reaction mechanism leading to Cl_2_^●−^ formation in aqueous solutions and involving ^●^OH radicals, HOCl^●−^ radical anions, protons (H^+^) and chlorine atoms (Cl^●^) [51]. The primary step of Met oxidation by Cl_2_^●−^ radicals was found to be, in principle, similar to that established for the ^●^OH radicals and occurs with absolute rate constant *k* = 3.9 × 10^10^ M^−1^ s^−1^ (see Table 1) [52]. It is characterized by a primary attack on the sulfur atom that constitutes a substitution of a chloride anion (Cl^−^) in Cl_2_^●−^ by Met and leads to the MetS∴Cl three-electron bonded species. Interestingly, from the earlier studies on the oxidation of simple thioethers it is known that MetS∴X species are involved in the following equilibrium with (MetS∴SMet)^+^ radical cations: MetS∴X + MetS ⇆ (MetS∴SMet)^+^ + X^−^ [53].

**Scheme 3:**
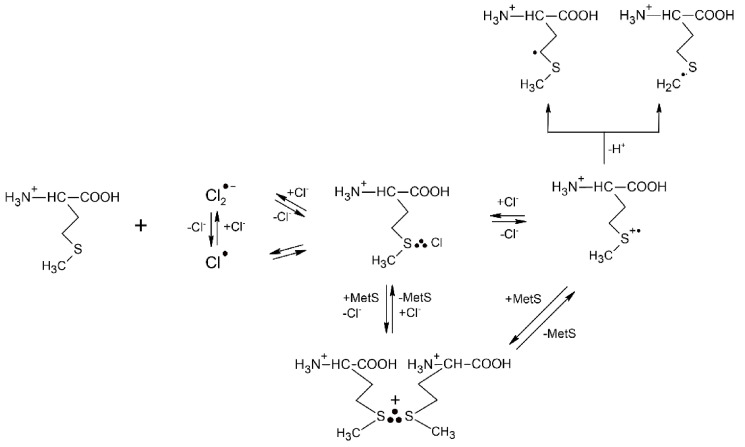
The reactions mechanism for Cl_2_^●−^−induced oxidation of methionine in aqueous solutions (based on [52]).

The reactions mechanism for Cl_2_^●−^-induced oxidation of Met is presented in Scheme 3 and clearly shows that a monomeric sulfur radical cation MetS^●+^ can be stabilized not only in a form of the dimeric (MetS∴SMet)^+^ radical cations but also in a form of MetS∴Cl three-electron bonded species with the chloride anions Cl^−^ providing a free electron pair. The validity of the mechanism (Scheme 3) was also experimentally demonstrated in the oxidation of Met by Br_2_^●−^ where the corresponding MetS∴Br species were also observed at neutral and basic pHs. The observed decrease in rate constant for the reaction of Met with Br_2_^●−^ (*k* = 2.5 × 10^10^ M^−1^ s^−1^) correlates well with the standard reduction potential of the Br_2_^●−^/2Br^−^ redox couple which is equal to E^0^ = +1.63 V vs. NHE (see Table 1) [47]. The actual reaction routes are very sensitive to a variety of parameters such as pH, Met and Cl^−^/Br^−^ concentration.

**Table 1:** Rate constants of reactions of ^●^OH radical and various one-electron oxidants (^●^Ox) with methionine in aqueous solutions.

Oxidant	Met(NH_3_^+^,COOH)	Met(NH_3_^+^,COO^−^)	Met(NH_2_, COO^−^)	E^0 (a)^	Lit
^●^OH	2.3 × 10^10 (b)^			+2.72	[7]
CCl_3_O_2_^●^			2.9 × 10^7 (b,c)^	+1.60	[45]
CF_3_ CHClO_2_^●^			1.4 × 10^6 (b,c)^	+1.15	[45]
CO_3_^●−^		3.6 × 10^7 (b)^		+1.57	[50]
Cl_2_^●−^	3.9 × 10^9 (b)^			+2.13	[52]
Br_2_^●−^	2.5 × 10^9 (b)^	1.7 × 10^9 (b)^	2.0 × 10^9 (b,c)^	+1.63	[52]
Tl^2+^		2.5 × 10^9 (b)^		+2.23	[43]
Ag^2+^		3.3 × 10^8 (b)^		+1.98	[43]

^(a)^ all reduction potentials in V vs. NHE after reference [47], ^(b)^ rate constants in M^−1^s^−1^ units, ^(c)^ measured by competition method.

#### 2.1.2. Methionine Derivatives

Selective protection of the N-terminal and C-terminal groups in Met enabled investigators to elucidate the role of each individual neighboring group NH_3_^+^/NH_2_ and COOH/COO^−^ in one-electron oxidation of Met by ^●^OH radicals. The following methionine derivatives were included in the studies: N-acetyl-methionine (N-Ac-Met) [7,54,55], methionine methyl ester (Met-C(=O)CH_3_ [54,56], and methionine ethyl ester (Met-C(=O)-CH_2_CH_3_ [46]. 

Substitution of one of the hydrogen atoms in the amino group (-NH_2_) by an acetyl group (-C=O)-CH_3_) does not change the initial reaction of ^●^OH radicals with the sulfur atom in N-Ac-Met. The observed transient absorption bands at pH 7 with λ_max_ = 290, 340, and 480 nm were respectively assigned to α-S1/α-S2 derived from N-Ac-Met, N-Ac-MetS∴OH, and (N-Ac-MetS∴SMet-N-Ac)^+^ species. However, the following features distinguish N-Ac-Met from Met: (i) prolonged lifetime of N-Ac-MetS∴OH, and (ii) absence of the absorption band which can be assigned to N-Ac-Met(S∴N)^+^. Based on these observations, it can be concluded that the protonated amino group in Met plays a role of intramolecular proton donor in the disappearance of MetS∴OH, and later being in the deprotonated form (-NH_2_) as a donor of free electron pair for stabilization of Met(S∴N)^+^. Depending on N-Ac-Met concentration, the monomeric sulfur radical cation N-Ac-MetS^●+^, similarly as in Met, can be stabilized via intermolecular equilibration with a second unattacked N-Ac-Met molecule forming a dimeric sulfur radical cation with intermolecular 2c-3e S∴S bond ((N-Ac-MetS∴SMet-N-Ac)^+^) and further undergo deprotonation to α-S1/α-S2 radicals (see Scheme 1). Detection of CO_2_ in the system studied [7,44], clearly indicates that N-Ac-MetS^●+^ can undergo decarboxylation via pseudo-Kolbe mechanism to form N-Ac-substituted α-N radicals [57,58].

The replacement of the carboxyl group with an ester or an amide group in methionine does not significantly change the primary radical reactions related to the oxidation process, except for the fact that the decarboxylation process involving the intramolecular Met(S∴N)^+^ is inhibited (see Scheme 1). This is reflected in much longer lifetime (τ_1/2_~1.1 ms) of this radical, which was directly observed in the example of the methionine ethyl ester [46].

Simultaneous acetylation of the N-terminal amino group and either esterification or amidation of the C-terminal carboxyl group was aimed at elimination of the fast intramolecular proton transfer from the amino group to the MetS∴OH moiety and elimination of decarboxylation of the Met residue via pseudo Kolbe mechanism, respectively. In addition, the insertion of these groups in the methionine molecule allows formation of structural motifs that quite well mimic peptide bonds in peptides and proteins. Two methionine derivatives, N-acetyl methionine amide (N-Ac-Met-NH_2_) [10], and methyl ester of N-acetyl-methionine (N-Ac-Met-OCH_3_) [11,59], were selected in order to limit stabilization of the monomeric sulfur radical cations (MetS^●+^) only to interactions with N- and O-atoms located in acetyl, ester and amide functional groups. Furthermore, they allow one to establish and to prove the mechanisms for primary and secondary radical reactions following oxidation of the Met residue by ^●^OH radicals with no contribution of free terminal amino and carboxyl groups. Thus, they can be considered to be the simplest models of Met residue incorporated in the interior of oligopeptides and proteins. The general reaction mechanism describing the primary and secondary reactions of radiationally induced ^●^OH radicals with these Met derivatives in aqueous solutions together with the respective intermediates is presented in Section 2.2.2. 

### 2.2. Photo-Induced Oxidation

**Scheme 4:**
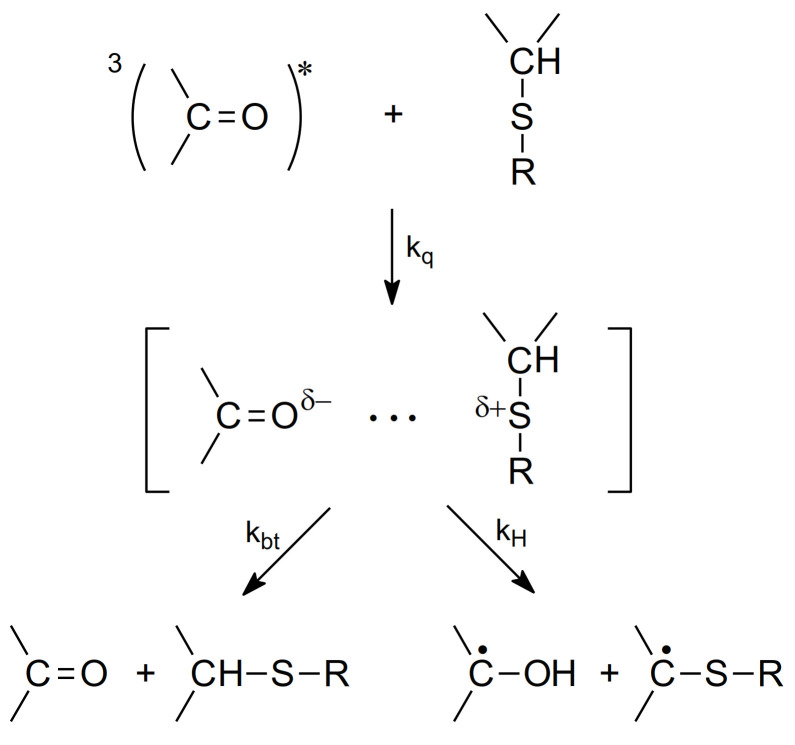
The mechanism for the quenching of ^3^Bz by thioethers (based on [60]).

The mechanism for the quenching of the benzophenone triplet state (^3^Bz) by sulfur-containing organic compounds, e.g., thioethers, has been studied since the 1970s [60]. It was suggested that quenching leads to the formation of partial charges on sulfur and oxygen atoms (Charge Transfer (CT)-complex) decaying by the proton transfer from the carbon atom bonded to the sulfur atom to form the ketyl radical and α-(alkylthio)alkyl radical (αS) and back electron transfer to regenerate reactants in their ground states (see Scheme 4).

To analyze the mechanism of quenching of carboxybenzophenone (CB) triplets by methionine, methionine derivatives and peptides (S and N atoms as the expected electron donors), one has to take into account that methionine can exist in zwitterionic (at pH 7) or anionic forms (at pH 11) in aqueous solutions depending on the pH range (see Figure 1). Therefore, the quenching rate constants for quenching the CB triplet in aqueous solutions by methionine, and Met derivatives and for comparison simple Met-containing peptides (Table 2) are presented at two representative pH values 7 and 10/11.

It was proven that the CB triplet quenching by methionine and Met-containing compounds were occurring via an electron transfer mechanism from its S-atom (not its N-atom) based on the following arguments [18,61]:

- as shown in Table 2, the quenching rate constants were found to be in the range of 10^9^ M^−1^ s^−1^ (diffusion controlled limit) for methionine and Met-containing compounds and were, respectively, three orders or one order of magnitude lower for amino acids without a sulfur atom (e.g., alanine) at pH 7 and pH 10,
- direct observation of radical-ion products in the transient absorption spectra (electron transfer intermediates, among them various two-center three-electron (2c-3e) bonded species) were products of oxidation of Met and CB radical anions and CB ketyl radicals were products of CB reduction,
- indirectly by the Rehm-Weller correlations of *k*_q_ vs. ΔG_el_ (free energy change for electron transfer).

Similar arguments for an electron transfer mechanism of quenching were applied for methionine derivatives with blocked amino and/or carboxylic terminal groups and simple peptides containing methionine residues [16,18,19,22,62,63].

**Table 2:** Quenching rate constants (*k*_q_ × 10^−9^ M^−1^ s^−1^) for quenching of CB triplet in aqueous solutions by methionine, Met derivatives and simple Met-containing peptides [15,16,18,19,20,62].

Amino Acid or Peptide	pH 7	pH 10/11
Methionine	2.5	2.3
N-Ac-Met	1.9	1.6
Met-OCH_3_	3.0	2.9
Met-Gly	2.1	2.3
Gly-Met	2.0	2.0
Met-Lys	1.8	1.3
Lys-Met	2.5	1.8
Met-Gly-Gly	2.3	2.2
Gly-Gly-Met	1.8	1.9
Met-Met	2.9	1.8
Met-Enkephalin	1.9	1.8
Alanine	<0.0005	0.18

estimated errors: <10%.

#### 2.2.1. Methionine

**Scheme 5:**
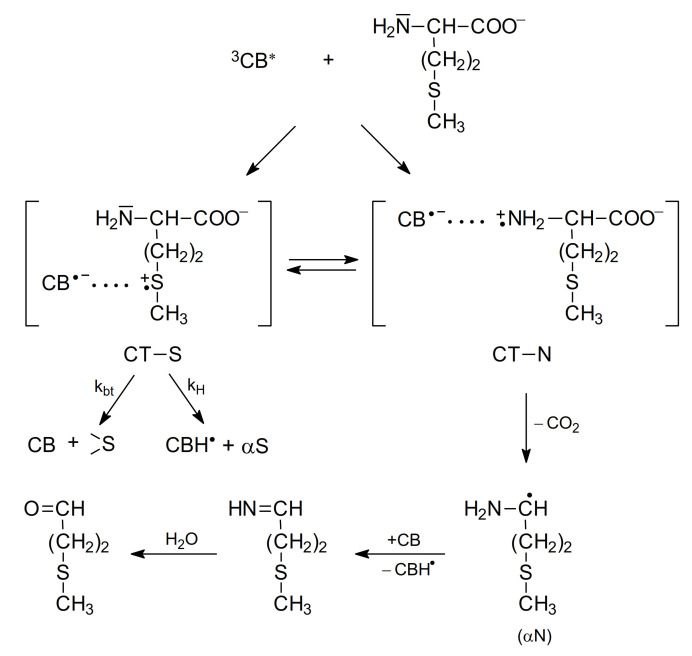
The mechanism of CB-sensitized photooxidation of methionine in alkaline aqueous solutions (based on [64]).

The study of the photooxidation mechanism of methionine in aqueous solution started with the pioneering work of Cohen [64]. The suggested mechanism of primary reactions was described by formation of two CT complexes: CT-S [CB^•−^…>S^•+^] and CT-N [CB^•−^…>N^•+^] but only CT-N complex could undergo decarboxylation leading to the formation of α-amino-alkyl radical (αN). The latter could reduce the ground state CB yielding ketyl radical (CBH^•^) and methional (Scheme 5). 

**Scheme 6:**
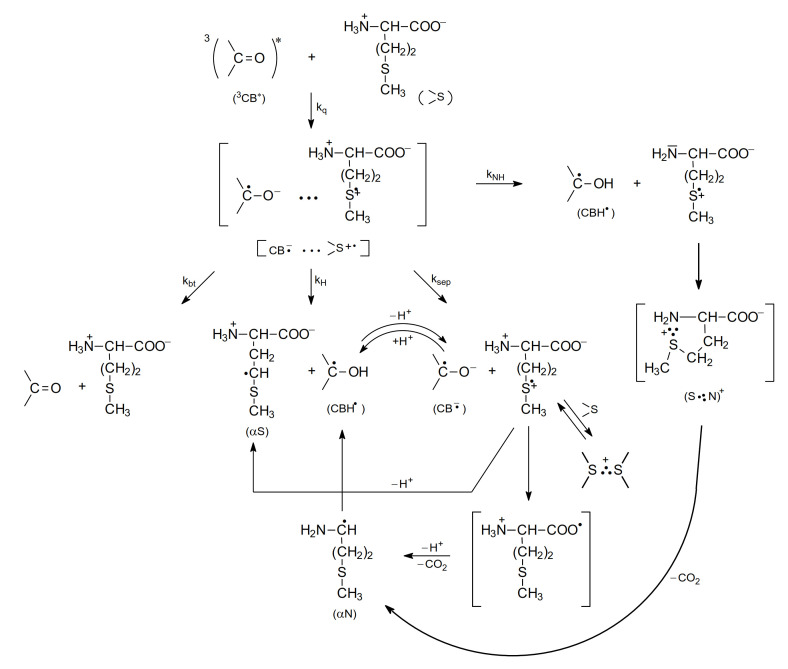
The mechanism of CB-sensitized photooxidation of methionine in neutral aqueous solutions (pH~7).

**Scheme 7:**
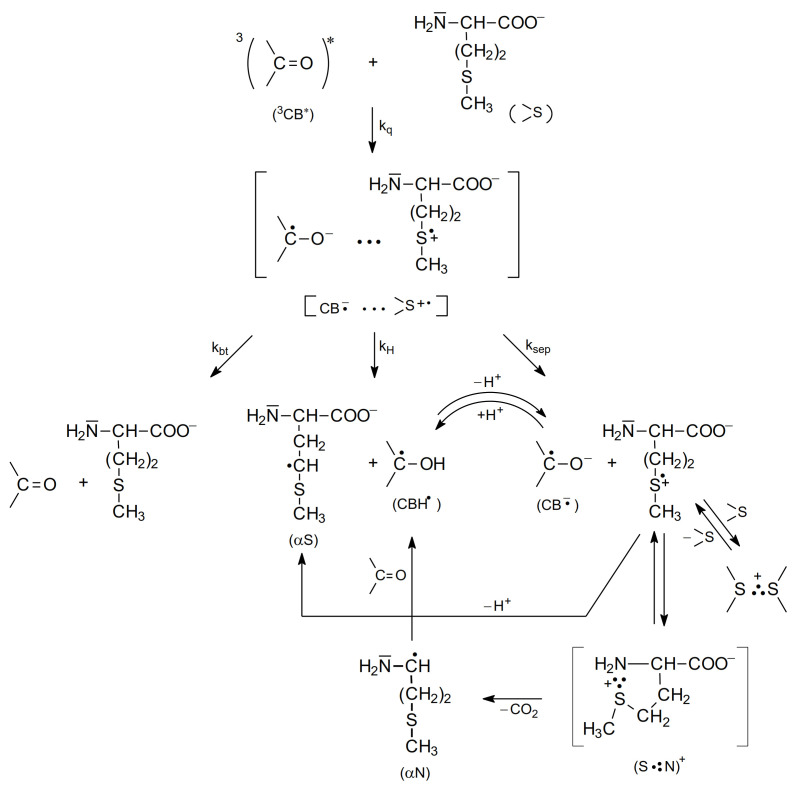
The mechanism of CB-sensitized photooxidation of methionine in basic aqueous solutions (pH~11).

Further studies using nanosecond flash photolysis technique [15,62,65,66] led to a more detailed description of primary and secondary reactions for the photooxidation of methionine in neutral and basic aqueous solutions. These reactions are presented in Scheme 6 and Scheme 7.

As presented in Scheme 6 (for methionine with the protonated amino group, pH 7), the electron transfer quenching of the CB triplet led to formation of a CT complex [CB^•−^…>S^•+^] that can decay in four main primary reactions: (1) charge separation (*k*_sep_) yielding CB^•−^ and >S^•+^ radical ions, (2) proton transfer within the CT complex (*k*_H_) yielding a ketyl radical CBH^•^ and an α-(alkylthio)alkyl radical (αS), (3) back electron transfer (*k*_bt_) leading to regeneration of the reactants in their ground states, and (4) proton transfer from the protonated amino group (-NH_3_^+^) to the CB^•−^ radical anion within the CT complex (*k*_NH_) leading to the ketyl radical CBH^•^ and sulfur-centered radical cation >S^•+^ with an -NH_2_ group (unprotonated) yielding a five-membered cyclic two-center three electron-bonded (S∴N)^+^ radical cation that can easy decarboxylate to form α-aminoalkyl radicals (αN). These short-lived intermediates can undergo further reactions. The >S^•+^ radical cations can be (i) stabilized by formation of three electron bonds with N, or S atoms (intramolecular (S∴N)^+^ and/or intermolecular (S∴S)^+^ in the reaction of >S^•+^ with >S reactant-methionine), (ii) deprotonated to form α-(alkylthio)alkyl radicals (αS), and (iii) decarboxylated to form α-aminoalkyl radicals (αN) via pseudo-Kolbe mechanism [57,58]. The latter radicals (αN), which in general can be formed in two reaction channels, can be involved in further reactions with the CB ground state leading to the formation of ketyl radicals and an imine [15,18,64]. Evidence for this hypothesis was a secondary growth of CBH^•^ in neutral or CB^•^^−^ in basic solutions that was observed after the CB triplets had decayed and that was also dependent on the concentration of CB. These observations indicated an additional reaction mechanism for CB reduction. This interpretation was supported by complementary pulse radiolysis experiments, where αN radicals were generated and their reaction with the CB in the ground state was studied [15].

The mechanism of photooxidation of methionine in basic aqueous solutions (methionine with a deprotonated amino group) presented in Scheme 7 is similar to the mechanism for neutral solutions (Scheme 6). However, as one would expect, the *k*_NH_ reaction is not present and the decarboxylation is due to a single reaction channel occurring via a very short-lived (S∴N)^+^ radical cations (several ns) (Scheme 7) [67].

The primary intermediates presented in Scheme 6 and Scheme 7 were observed directly using time-resolved techniques (that included the kinetics of their formations and decays), e.g., nanosecond laser flash photolysis [15,18,67]. The transient absorption spectra obtained in these experiments were resolved at various time delays into the component spectra using a spectral-resolution procedure. This procedure, together with the reference spectra of the expected transients, were described in detail in reference [68]. As a final result of this analysis, the concentrations of all of the transients were determined at various time delays following the laser pulse and were presented as concentration profiles of the transients. Thus, the details of the primary and secondary reactions of CB-sensitized photooxidation of methionine and its derivatives (including formation and decays of intermediates) could be followed by laser flash photolysis techniques. Unfortunately, due to the ground state absorption of the CB sensitizer, the spectral window available for nanosecond laser flash photolysis studies was limited to λ > 360 nm. As a consequence, some intermediates, including αS–α-(alkylthio)alkyl radical with its maximum absorption at λ = 290 nm, could not be observed directly. 

Using a relative actinometry method with a CB solution as an external actinometer (the absorbances at the excitation wavelength of CB both in the reaction cell and in the actinometer cell, were identical), the initial quantum yields for formation of the intermediates were determined (for experimental details see [68]). The reaction of α-aminoalkyl radicals (αN) with the CB in the ground state led to the slow secondary formation of ketyl radicals (CBH^●^) or ketyl radical anions (CB^●−^) depending on pH and its efficiency can be described as Φ”CBH^•^ or Φ”CB^•−^. The quantum yields measured for aqueous solutions at pH~7 and pH~11 for primary and secondary intermediates are presented in Table 3.

Time-resolved and steady-state studies for the CB-sensitized photooxidation of Met (Table 3) led to the following conclusions regarding the primary photochemical reactions of methionine at neutral pH (see Scheme 6): (i) the charge separation reaction is an efficient process yielding the ketyl radical anion (CB^•−^) and the sulfur-centered radical cation >S^•+^ with quantum yield Φ_CB•−_ = 0.27, (ii) >S^•+^ radical cations can react (reversible reaction) with the excess of methionine in its ground state leading to the intermolecular (S∴S)^+^ dimeric sulfur radical cations with Φ_(S__∴S)+_ = 0.23, approximately equal to the value of the quantum yield of charge separation (Φ_CB•−_), (iii) *k*_NH_ proton transfer within the CT complex led to ketyl radicals CBH^•^ and CO_2_ as final products with quantum yields ≤ 0.11 (Φ_CBH•_), (iv) decarboxylation reaction >S^•+^ radicals via pseudo-Kolbe reaction [57,58] cannot be neglected. 

In the case of methionine in basic solutions (Scheme 7), the main primary reaction is the charge separation reaction (with Φ_CB•−_ = 0.66 twice that for neutral pH) and the *k*_H_ primary reaction is more than ten times less efficient than *k*_sep_ reaction. Moreover, the intermolecular (S∴S)^+^ intermediate is present along with an efficient decarboxylation (Φ_CO2_ = 0.55) occurring via a very short-lived intramolecular (S∴N)^+^ radical cation (ns time scale).

As shown in Table 3, the quantum yields for secondary reactions at pH 7 and pH 11 (Φ”_CBH•_ or Φ”_CB•−_) are equal within the experimental errors with quantum yields of decarboxylation (Φ_CO2_ = 0.28 or Φ_CO2_ = 0.55, respectively). However, the mechanism for the slow formation of the ketyl radical anion CB^•−^ in the secondary reactions was shown to be very complex (e.g., Φ_CB•−_ was pH dependent and decrease for pH above 11 and at pH 13.2 was approaching zero). This was explained by the involvement of additional acid-base equilibria for sulfur- and nitrogen-centered radical cations, see reference [67]. 

In addition to the time-resolved kinetic studies using nanosecond laser flash photolysis, the mechanistic conclusions, which are presented in Scheme 6 and Scheme 7 for methionine, time-resolved CIDNP, and ESR techniques, have expanded our mechanistic understanding of radical reaction pathways of the Met side chain in various local environments containing differing functional groups. The CIDNP study performed by Goez et al. [23,69] for CB-sensitized photooxidation of methionine confirmed the presence of an electron transfer mechanism involving the sulfur atom (not the nitrogen atom) as the electron donor. The formation of a two-center three electron bonded radical cation (S∴N)^+^ in alkaline solutions (pH = 12) and the presence of the monomeric sulfur-centered radical cation >S^•+^ in neutral solution (pH = 6) were also proved. 

A time-resolved ESR spectroscopy study by Yashiro et al. [26] for anthraquinone-sulfonate sensitized photooxidation of methionine in aqueous solution led to the following conclusions: at low pH the intermolecular dimeric sulfur radical cation (S∴S)^+^ was the main intermediate but at high pH, deprotonation of a monomeric sulfur-centered radical cation >S^•+^ to form the aminyl radical (CH_3_-S-CH_2_-CH_2_-CH-(COO-)-N^•^H) was suggested as the main reaction channel. However, the α-(alkylthio)alkyl radicals (αS) were not detected in their ESR experiments.

Some differences in the description of the mechanisms and main intermediates were observed for Met at high pH i the results of ESR by Yashiro et al. [26] and the nanosecond laser flash photolysis studies could be due to differences (i) in the sensitizers used in these two types of experiments (anthraquinone sulfonate (AQS) vs. 4-carboxybenzophenone (CB)) and (ii) in the experimental conditions (e.g., laser pulse energies 90 mJ in comparison with 1–6 mJ, respectively). This may lead to differences in the decay channels of the respective CT complexes [Sens^•−^…>S^•+^] with AQS and CB as sensitizers. Moreover, the spectral window available in the nanosecond laser flash photolysis did not allow detection of aminyl radical (CH_3_-S-CH_2_-CH_2_-CH-(COO-)-N^•^H) observed in the EPR experiments. Furthermore, as was presented by Bonifačić et al. [70] that type of radicals can undergo further transformation via 1,2-H shift to α-C-centered radicals. On the other hand, identification of radical-radical coupling products such as αS-αS in the steady-state photooxidation of Met derivatives sensitized by CB [22,59] confirmed the presence of αS radicals in the reaction mechanisms.

The mechanism for the photooxidation of methionine was also studied using sensitizers different from CB, namely N-(Methylpurin-6-yl)pyridinium cation (Pyr^+^) in aqueous solution at neutral pH [71]. It was shown by nanosecond laser flash photolysis and steady-state photolysis studies that the mechanism of photooxidation is, in general, similar to that for CB-sensitized photooxidation of methionine at pH 7 [15]. The quenching of Pyr^+^ excited triplet state led to the decay of the CT complex [Pyr^•^…>S^•+^] in two primary reactions: (i) charge separation to a Pyr^•^ radical and an >S^•+^ radical cation and (ii) back electron transfer to regenerate reactants in their ground states. The reactions of the resulting sulfur-centered radical cation >S^•+^ are similar to those seen in CB-sensitization (see Scheme 6). These radical cations can undergo decarboxylation yielding an α-aminoalkyl radical (αN) that can reduce Pyr^+^ cations to additional Pyr^•^ radicals (slow secondary growth of Pyr^•^) and formation of intermolecular (S∴S)^+^ dimeric sulfur radical cations in reaction to methionine in its ground state. The time-resolved and steady-state studies led to a detailed quantitative description of the primary and secondary reactions for methionine and other sulfur-containing amino acids [71]. 

#### 2.2.2. Methionine Derivatives 

**Scheme 8:**
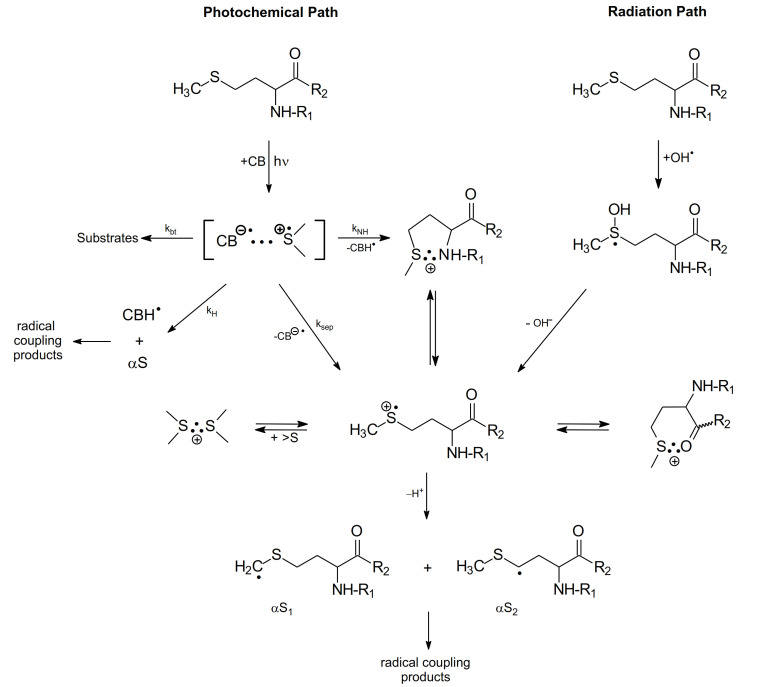
The mechanism of CB-sensitized photo- and radiation-induced oxidation of methionine derivatives in aqueous solutions (R_1_ denotes N-Ac- group or H- and R_2_ denotes -OCH_3_, -NH_2_ and NHCH_3_ groups).

In this subsection, the methionine derivatives, the methyl ester of N-acetyl-methionine (N-Ac-Met-OCH_3_) and the N-methylated amide of N-acetyl-methionine (N-Ac-Met-NHCH_3_) were selected as simple models of a Met residue incorporated into the interior of oligopeptides and proteins (Scheme 8). 

The purpose for acetylating the N-terminal amino group was to eliminate the fast intramolecular proton transfer from the amino group to the >S∴OH moiety (see Section 2.1.2). A similar purpose for acetylating the N-terminal amino group applies to the CB-sensitized photooxidation of Met. In this case, fast intramolecular proton transfer from the N-terminal amino group of Met to the ketyl radical anion (CB^●−^) within the [CB^•−^…>S^•+^] complex [19], (see *k*_NH_ reaction channel, in Scheme 6) is eliminated. On the other hand, esterification of the C-terminal carboxyl group or amidation by the N-methylated amide group (-NHCH_3_) eliminates decarboxylation of Met residue via the pseudo-Kolbe mechanism [57,58]. Therefore, the use of N-Ac-Met-OCH_3_ or N-Ac-Met-NHCH_3_ has the following benefit: it allows one to mimic and prove the mechanisms for primary and secondary radical reactions following oxidation of the Met residue with no contribution of N- and C-terminal functional groups. 

The results of intensive studies for photo- and radiation-induced oxidation of methionine derivatives (with blocked amino and carboxylic groups) by the complementary laser flash photolysis and pulse radiolysis techniques are summarized in Scheme 8, showing primary photo- and radiation-induced processes.

As presented in Scheme 8, both the photochemical and radiation pathways led to the formation of one common intermediate, namely the sulfur-centered radical cation (>S^•+^). In the case of photochemical pathway the formation of (>S^•+^) is occurring via one of the four competing primary reactions of CT complex, namely the charge separation reaction of [CB^•−^… >S^•+^]. For the radiation pathway, a main channel leading to (>S^•+^) is the HO^−^ release from the MetS∴OH adduct. These pathways exemplify neighboring group participation in the reactions of two intermediates, the sulfur radical cations (>S^●+^) and MetS∴OH during Met oxidation (see Scheme 8) [37]. As was mentioned in the previous sections, two-centered, three electron (2c-3e) bonds between the oxidized sulfur atom and the lone electron pairs located on the nitrogen atom in the N-terminal amino group and the oxygen atoms in the C-terminal carboxyl group are responsible for stabilization of >S^●+^. Such stabilization leads to five-membered intramolecularly S∴N-bonded species and/or six-membered intramolecularly S∴O-bonded species [8,9,16,17,18,19]. Subsequent studies showed that heteroatoms present in the peptide bond can be also involved in the formation of similar transient species with 2c-3e bonds with the oxidized sulfur atom [11,12,13,72,73,74]. 

The secondary reactions of intermediates shown in Scheme 8 led to formation of various stable products. According to our knowledge, there were only two attempts to identify stable products after HO^●^ or CB-induced oxidation of methionine derivatives [22,59]. They addressed the full mechanism of Met oxidation from the initial step (hν excitation or radiolysis) via identification of short-lived intermediates (by time-resolved techniques) to the analysis of stable products (in steady-state UV irradiation or γ-radiolysis). Knowledge of the structure of stable products was a strong argument confirming the assignments of intermediates detected in the time-resolved experiments.

Quantum yields of primary intermediates in the CB-sensitized photooxidation of Met derivatives are summarized in Table 4. In the case of methionine derivatives with a blocked carboxylic group (MetOCH_3_ and MetNH_2_) (S∴N)^+^ was the main primary intermediate regardless of pH values. At low pH, where amino group is protonated (see Figure 1), the presence of the (S∴N)^+^ radical cation can be rationalized via the k_NH_ reaction (Scheme 8). For N-Ac-Met: (i) the charge separation reaction (k_sep_) is the main reaction of the CT complex [CB^∙−^…>S^∙+^] decay, (ii) the decarboxylation reaction can be neglected and (iii) the intermolecular dimeric sulfur radical cation (S∴S)^+^ is the main intermediate. For methionine derivatives with blocked amino and carboxylic groups (N-Ac-Met-OCH_3_, N-Ac-Met-NHCH_3_ and N-Ac-Met-NH_2_), the proton transfer within the CT complex (k_H_) yielding ketyl radical CBH^∙^ and α-(alkylthio)alkyl radical (αS) was found to be the dominate primary reaction over the charge separation reaction (k_sep_) that yielded only a small amount of CB^∙−^ and (S∴S)^+^ dimer. The presence of these intermediates was confirmed by the analysis of stable products formed during the steady-state photolysis. The main stable products were found to be the combination products of ketyl radicals with α-(alkylthio)alkyl radical (αS-CBH stable products) [22,59]. 

An additional argument for a mechanism of the proton transfer reaction within the CT complex (k_H_) for methionine derivatives with substituted amino and carboxylic groups came from the photochemical study of benzophenone-methionine dyads (diketopiperazine-based BP-Met) in acetonitrile solutions [76]. Both the time-resolved laser flash photolysis experiments and the steady-state irradiations of sterically constrained BP-Met dyads identified an electron transfer process from the sulfur atom to the BP triplet followed by proton transfer to form biradical (with quantum yields in the range of 0.6). This biradical was a major precursor of a cyclic stable product (an analogous product to αS-CBH observed in the photolysis of N-Ac-MetOCH_3_ and N-Ac-MetNHCH_3_).

## 3. Methionine in Linear Peptides

### 3.1. Radiation-Induced Oxidation

#### 3.1.1. Methionine as the N/C-Terminal Amino Acid Residue

The location of the Met residue in the linear peptide structure was found to affect the type of intermediates formed during ^●^OH-induced oxidation. This was demonstrated for simple dipeptides containing Met with the reversed sequence of amino acids: Met-Gly/Gly-Met and Met-Leu/Leu-Met [8,77]. For Met-Gly and Met-Leu dipeptides the observed transient absorption bands in the pH range 5—6 with λ_max_ = 290 and 390 nm were respectively assigned to α-S1/α-S2 radicals derived from Met and Met(S∴N)^+^ radical cations. Thus, the location of Met residue as the N-terminal amino acid does not significantly change the primary radical reactions related to the oxidation process (see Scheme 1), except for the fact that the decarboxylation process involving the intra-molecular Met(S∴N)^+^ is inhibited, although the carboxyl group in C-terminal Gly or Leu residues is not protected. This is reflected in a much longer lifetime (τ_1/2_~200—400 μs) of these radical cations and lack of presence of CO_2_ in the system containing Met-Gly [57]. Interestingly, in the presence of superoxide radical anion (O_2_^●−^) this species decays much faster with the rate constant k(Met(S∴N)^+^ + O_2_^●−^) = 5.3 × 10^9^ M^−1^ s^−1^ [78]. It is important to note that the reaction of O_2_^●−^ with superoxide dismutase (SOD) with the respective k = 2.3 × 10^9^ M^−1^ s^−1^ proceeds ca. 2.5-fold slower than its reaction with Met(S∴N)^+^ [79]. From biological point of view it means that reaction of Met(S∴N)^+^ with O_2_^●−^ is a potential source for methionine sulfoxide formation when the system is exposed to high concentration of reactive oxygen species (ROS). 

**Scheme 9:**
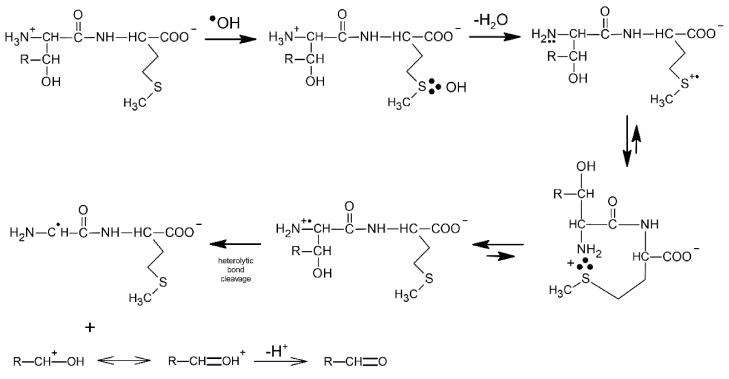
General reaction scheme for ^●^OH-induced oxidation of methionine in Ser-Met and Thr-Met dipeptides (R = H for serine, R = CH_3_ for threonine).

**Scheme 10:**
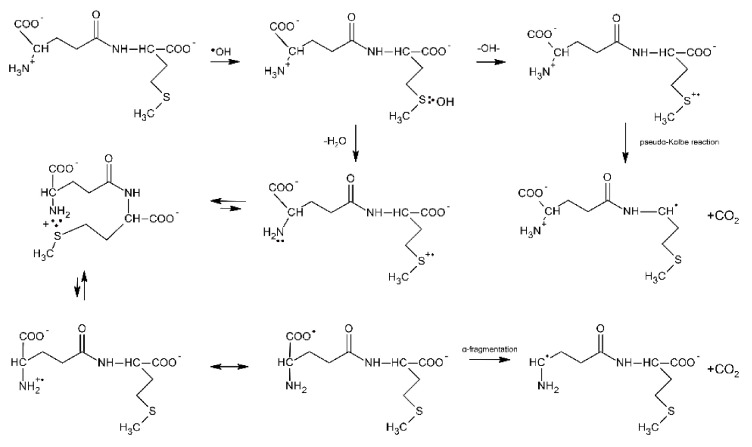
General reaction scheme for ^●^OH−induced oxidation of methionine in γ-Glu-Met dipeptide.

**Scheme 11:**
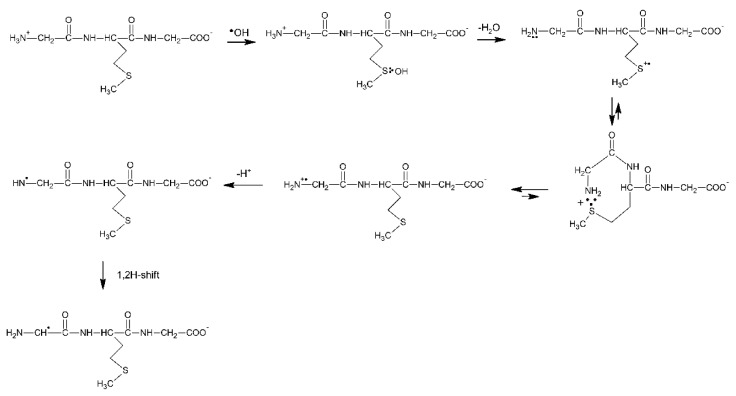
General reaction scheme for ^●^OH−induced oxidation of methionine in Gly-Met-Gly tripeptide.

On the other hand, for Gly-Met and Leu-Met dipeptides, the observed transient absorption spectrum in the same pH range is dominated by the absorption band with λ_max_ = 290 nm and a clearly pronounced shoulder in the 360–400 nm range. These absorption bands were assigned to α-S1/α-S2 radicals derived from Met and Met(S∴O)^+^ radical cation, respectively. Taking into account the character of intermediates and final products detected during ^●^OH-induced oxidation of dipeptides containing serine, threonine and γ-glutamic acid as the N-terminal amino acids (see Scheme 9 and Scheme 10), it is reasonable to assume that fast intramolecular proton transfer from the free amino group (NH_3_^+^) of the dipeptide to the MetS∴OH moiety occurs also for dipeptides containing amino acids with alkyl side groups. The sulfur monomeric radical cation (MetS^●+^) resulting from this reaction can decay along three different pathways: (i) forming the very-short-lived multi-membered S∴N-bonded radical cation which can be the precursor of C-centered radicals (on the α-C atom of the Gly residue) formed via analogous consecutive reactions presented in Scheme 11, (ii) forming the Met(S∴O)^+^ radical cation, (iii) undergoing decarboxylation via the pseudo-Kolbe mechanism to form substituted α-N radicals on the Met residue confirmed by detection of CO_2_ [57]. 

The first convincing evidence for intramolecular proton transfer from the free amino group (NH_3_^+^) of the dipeptide to the MetS∴OH moiety and formation of the multi-membered S∴N-bonded radical cation was obtained from the studies of ^●^OH-induced oxidation of Ser-Met and Thr-Met dipeptides [14]. The general reaction scheme involves an intramolecular proton transfer from the protonated N-terminal amino group to an initially formed MetS∴OH radical on the Met residue. The MetS∴OH undergoes subsequently elimination of water and formation of a Met(S∴N)^+^ intermediate which was characterized by pulse radiolysis and has a lifetime (τ_1/2)_ ≈ 300 ns). This intermediate exists in an equilibrium with the open chain N-centered radical cation which further undergoes efficient heterolytic bond cleavage of the C_α_−C_β_ bond of the Ser or Thr side chain, leading to an α-aminoalkyl radical of the structure H_2_N-C^●^H-C(=O)-NH-peptide (identified by time-resolved ESR spectroscopy) and formaldehyde or acetaldehyde, respectively (Scheme 9). 

The above mechanism illustrates an interesting feature which has to be considered in any studies on oxidation of peptides containing Met. Although, based on the respective rate constants, Met is the main target of ^●^OH radicals attack, the major final product contains fragments of either Ser or Thr residues. Such transfer of damage constitutes an important mechanistic pathway frequently observed during oxidation of peptides and proteins.

Further evidence of proton transfer from the free amino group of the peptide to the MetS∴OH moiety and formation of the multi-membered S∴N-bonded radical cation was obtained from studies of ^●^OH-induced oxidation of the γ-Glu-Met dipeptide [80]. The mechanism is in principle very similar to that presented for Ser-Met and Thr-Met except for the fact that the open chain N-centered radical cation via establishment of the mesomeric form (H_2_N-CH-(COO^●^))-peptide and subsequent α-fragmentation leads to α-aminoalkyl type radicals and CO_2_ (Scheme 10). 

These radicals obtained from the N-terminal decarboxylation reaction can be easily probed via their reaction with p-nitroacetophenone (PNAP) leading to PNAP^●−^ radical anion [80,81,82]. The following fact should be emphasized at this point: the radiation chemical yield of CO_2_ measured for this dipeptide was not equal to the radiation chemical yield of α-aminoalkyl type radicals [80]. This implies that decarboxylation must proceed via two different routes, namely via the Met(S∴N)^+^ intermediate and via a pseudo-Kolbe reaction. This finding differs from X-Met peptides (X = Gly, Ala, Val, Leu) where decarboxylation was found to occur exclusively via the latter mechanism [57]. Insertion of a Gly residue between the γ-Glu and Met residues in γ-Glu-Gly-Met-Gly peptide increases the separation distance through the bonds as compared with γ-Glu-Met; however, it does not affect the radiation chemical yield of α-aminoalkyl type radicals G(α-N) [81]. This implies that the yields of multi-membered S∴N-bonded radical cations G(S∴N)^+^ have to be similar, which at the same time means that the rate of end-to-end contact formation is similar in both peptides. In other words, the peptide backbone in γ- Glu-Gly-Met-Gly is flexible enough to allow direct interaction between amine and sulfur moieties. The γ-Glu-(Pro)_n_-Met (n = 0–3) oligopeptides were chosen as oligopeptides with restricted conformational flexibility [82]. The observed continuous decrease of G(αN) with the number of Pro residues (from 0 to 3) indicates that formation of a contact between the S-atom in the C-terminal Met residue and the N-atom of a deprotonated N-terminal amino group of Glu is controlled by the relative diffusion of the S^●+^ and unoxidized N-atom. This study clearly shows that a remote amino group that served as both a proton and a free electron pair donor and that is separated from the Met residue by an oligoproline rigid backbone can still affect the chemistry on Met.

It also worth mentioning that neighboring amino acids (such as tyrosine or tryptophan) can affect the chemistry on Met. Taking into account their respective reduction potentials (E^0^(TrpN^●^/TrpN) = +1.015 V vs. NHE at pH 7) [83], E^0^(TyrO^●^, H^+^/TyrOH = +0.93 V vs. NHE at pH 7) [84] and E^0^(MetS∴OH/Met,HO^−^) = +1.43 V vs. NHE, E^0^(MetS^●+^/MetS) = 1.66 V vs. NHE [85], they can be easily oxidized by the monomeric sulfur radicals MetS^●+^ or MetS∴OH adducts. This phenomenon was nicely illustrated with the example of Met-enkephalin. Met-enkephalin (Met-enk), is a part of a mixture with Leu-Enk and is present in the central nervous system. Met-enk is a pentapeptide: Tyr-Gly-Gly-Phe-Met and is produced while an organism is under mental and/or physical stress [86]. The degradation induced by ^●^OH radicals in Met-enk is also relevant to the disorders in inflammatory processes. 

Nanosecond pulse radiolysis was used to elucidate the oxidation mechanisms of Met-enk by ^●^OH radicals [87]. Based on the respective rate constants of Tyr, Phe, and Met residues with ^●^OH radicals [88], formation of OH adducts on both aromatic (Tyr, Phe) and Met residues is reasonable and expected. Interestingly, fast formation of OH adducts on Tyr and Phe residues was confirmed by pulse radiolysis; however, no transient which can be assigned to MetS∴OH was observed. Moreover, the fast formation of the transient spectrum assigned to TyrO^●^ radicals was also observed. For a comparison, the absorption spectra of transients derived from Leu-enk (the C-terminal Met is replaced by Leu) were recorded and assigned only to the formation of OH adducts on Tyr and Phe residues. These differences in absorption spectra led to the hypothesis that TyrO^●^ radicals are formed through a new process not present in Leu-enk, i.e., an intramolecular electron transfer (IET) involving a MetS∴OH intermediate and a Tyr residue. The lower limit for the rate constant of IET was found to be 1.2 × 10^7^ s^−1^. The high value for the rate constant for IET in Met-enk is consistent with the lack of α-(alkylthio)alkyl radicals and with the lack of CO_2_ formation [57]. Depending on the pH, the former radicals might be potentially produced by the competitive deprotonation of either MetS∴OH or MetS^●+^. In turn, the formation of CO_2_ would have resulted from the competitive oxidation of the carboxylate function in Met via a pseudo Kolbe mechanism. Similar oxidation studies of Met-enk were performed using Br_2_^●−^ as a selective one-electron oxidant. They allowed for the determination of the first order rate constant of an IET involving MetS∴Br and Tyr residues (k = 1.1 × 10^5^ s*^−^*^1^) [89]. On the other hand, the rate constant for IET between MetS∴Br and Tyr residues in Tyr-(Pro)_3_-Met (a peptide with the same number (3) of amino acid residues between Tyr and Met residues as in Met-enk) was found to decrease 10-fold and be equal to 1.1 × 10^4^s^−1^ [90]. In the latter case an IET is likely partitioning along the peptide backbone and direct water mediated contacts between side chains of terminal amino acids residues (Tyr and Met). Therefore, in Met-enk, an IET occurs most probably rather through space (water) pathway with a possible involvement of H-bonds shortcuts. This conclusion is consistent with computational conformational analysis of this pentapeptide [91]. At the end of these considerations, it has to be stressed that the possibility for fast IET between an oxidized Met residue and the easily oxidized Tyr and Trp residues may lead to repairing of Met oxidative damage, along with the formation of damage in other sites of a peptide or protein molecule. 

#### 3.1.2. Methionine as the Internal Amino Acid Residue

The Gly-Met-Gly peptide is the simplest model peptide where the Met residue is not a terminal amino acid. Therefore, this peptide can serve as the model of Met residues incorporated in the interior of oligopeptides and proteins. The reaction of ^●^OH radicals with Gly-Met-Gly and its N-acetyl derivative were studied by pulse radiolysis [74]. The transient absorption spectra recorded at short times after the pulse in N_2_O-saturated aqueous solutions at pH 5.5 were characterized by a strong UV absorption band with λ_max_ = 270 nm and a very weak and broad shoulder in the range of 400–500 nm. Spectral resolutions of these spectra were necessary in order to resolve them into contributions from various intermediates: the α-C-centered radicals on Met residues (α-C), the α-aminoalkyl radicals (α-N), the α-(alkylthio)alkyl radicals (α-S), the intramolecular Met(S∴N)^+^ radical cations and the intermolecular Met(S∴S)^+^ radical cations. Their radiation chemical yields and percentage contribution (in parenthesis) to the total yield of radicals formed in the reaction of OH is listed in Table 5. 

It is worthwhile to note that the contribution of MetS∴OH is neglected for short times after the pulse, which means that its lifetime is very short. This observation can be rationalized by analogy with peptides containing C-terminal Met (see Section 3.1.1), in terms of a concerted process which involves a fast proton transfer from the N-terminal -NH_3_^+^ group to the MetS∴OH moiety, which eliminates HO^−^ (in the form of water) occurs and leads by sequential steps to α-N radicals (Scheme 11). It has to be stressed at this point that α-N and α-S radicals are the most abundant radicals resulting from the ^●^OH-induced oxidation of Gly-Met-Gly and constitute more than 80% of all of the radicals. Despite the fact that the carboxyl group in Gly residue is free, the pseudo Kolbe reaction does not operate in this case which was confirmed by the lack of CO_2_ [57]. Acetylation of the N-terminal amino group in Gly-Met-Gly eliminates any fast proton transfer to the MetS∴OH moiety which is reflected in the longer lifetime of MetS∴OH radicals, and as a consequence, their substantial contribution at short times after the electron pulse (Table 6). 

Moreover, formation of the multi-membered S∴N-bonded radical cation (precursors of α-N radicals) is not possible and the sequential reaction steps leading to α-N radicals are switched off (Scheme 11). This is confirmed by the fact that α-N radicals were not necessary for inclusion in the spectral resolutions. 

**Figure 3:**
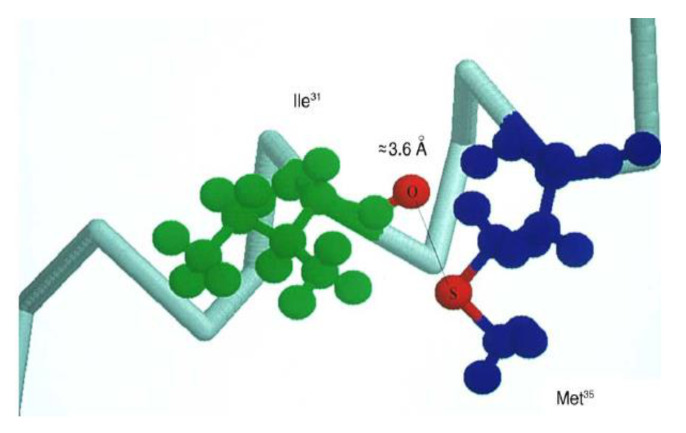
Schematic drawing of the α-helical portion of the C-terminal region that encompasses Met^35^ (reprinted with permission from [94]).

The first experimental proof that Met can be oxidized by radicals with the reduction potentials lower than +1.4–1.6 V vs. NHE (which is equal to E^0^(MetS^●+^/Met)) came from one-electron oxidation of β-amyloid peptide (β-AP1-40) using N_3_^●^ radicals with E^0^(N_3_^●^/N_3_^−^) = 1.33 V vs. NHE [92]. Thermodynamic considerations indicate that N_3_^●^ should not oxidize Met residues unless the one-electron reduction potential of Met is lowered because of favorable environment. It was shown that Met^35^ is the target in β-AP1-40 oxidation. Molecular modeling studies showed that β-AP26-40 (a representative fragment of the native β-amyloid peptide (β-AP1-42) has a large probability of forming S−O bonds between Ile^31^ and Met^35^ due to the specific structural properties of the polypeptide fragment (an α-helical motif) (Figure 3) [93,94]. These results corroborate the reported role of Met^35^ in the toxicity and protein oxidation capacity of β-AP(1–40) [95,96,97].

#### 3.1.3. Linear Peptides with Two Methionine Residues 

Contrary to cyclic dipeptides (see Section 4) extended conformers dominate in the linear (open chain) Met-Met dipeptides due to expressed tendency toward trans amides. Therefore, the close contacts between side chains of Met residues should be enhanced in D,L- and, L,D-stereoisomers in comparison to L,L- and D,D-stereoisomers (see Section 4.1.2). However, one should expect weaker steric constraints in linear peptides in comparison to cyclic dipeptides (see Section 4*).*

The reaction of ^●^OH radicals with four stereoisomers was investigated by pulse radiolysis with time-resolved UV-vis spectrophotometry in Ar or N_2_O saturated aqueous solutions at pH 1 and pH 5.2, respectively [9]. The absorption bands observed at pH 1 for D,L-, L,D-, L,L-,and D,D-stereoisomers were characterized by a maximum with λ_max_ = 490 nm and similar molar absorption coefficients ε_490_ = 5900, 5550, 5500, and 5800 M^−1^cm^−1^, and were unequivocally assigned to intramolecular Met(S∴S)^+^ radical cations. The similarity between the maximum positions and the molar absorption coefficients suggests similar overlap of p-orbitals of sulfur atoms and thus a similarity in the strength of the S∴S-bond in the radical cations derived from all of the optical isomers [98,99] and thus confirming weaker steric constrains in linear peptides than in cyclic dipeptides (see Section 4.1.2), whereas the influence of the stereoisomerism was manifested in the decay rates of Met(S∴S)^+^ radical cations: first order decay rate constants were measured at pH 1: 6.2 × 10^3^ s^−1^ (L,L-isomer), 8.3 × 10^3^ s^−1^ (D,D-isomer), 3.9 × 10^3^ s^−1^ (L,D-isomer), and 3.7 × 10^3^ s^−1^ (D,L-isomer). These Met(S∴S)^+^ radical cations are in an equilibrium with the respective monomeric sulfur radical cations (MetS^●+^) The observed differences can be understood in terms of different equilibrium constants (K_eq_) due to different distances between the NH_3_^+^ group and the positively charged S-atom. At pH = 5 the transient absorption observed for L-Met-L-Met was characterized by a strong absorption band with λ_max_ = 390 nm which was assigned inter alia to Met(S∴N)^+^ radical cation which are formed via dehydroxylation of the primary MetS∴OH radical at the N-terminal Met assisted by an intramolecular proton transfer from the NH_3_^+^ group. A weaker absorption band with λ_max_ = 290 nm was assigned to α-(alkylthio)alkyl radicals (α-S). Because of neutral character of ^●^OH radicals it is reasonable to assume that both sulfur atoms located in the N-terminal and C-terminal Met residues are attacked by ^●^OH radicals with the same efficiency. Dehydroxylation of MetS∴OH at the C-terminal Met can be also assisted by an intramolecular proton transfer from the NH_3_^+^ in the process analogous to Gly-Met-Gly tripeptide (see Section 3.1.2, Scheme 11). In this case, the monomeric sulfur cations (MetS^●+^) can form an intramolecular Met(S∴O)^+^ radical cation, deprotonate to α-S radicals, and undergo a pseudo Kolbe reaction that leads to decarboxylation. The last reaction can be excluded based on the photochemical experiments where L-Met-L-Met was oxidized by the triplet of 4-carboxybenzophenone (see Section 3.2.2) and no CO_2_ was detected.

**Scheme 12:**
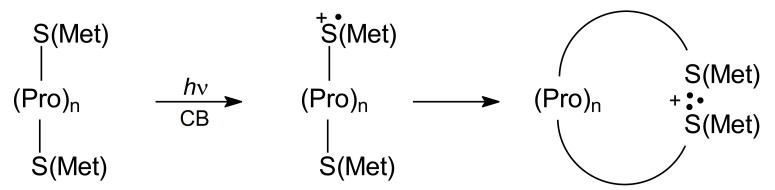
Formation of intramolecular dimeric sulfur radical cations (S∴S)^+^ in photo-induced oxidation of Met-(Pro)_n_-Met oligopeptides.

Similarly, as for the γ-Glu-(Pro)_n_-Met (n = 0–3) oligopeptides (see Section 3.1.1), the Met-(Pro)_n_-Met (n = 0–4) oligopeptides were chosen as oligopeptides with restricted conformational flexibility. The radiation chemical yields of intramolecular Met(S∴S)^+^ radical cations depend on the number of Pro-residues; however, they do not depend in a simple way on the average distance between the sulfur atoms in the Met residues. The analysis showed that formation of a contact between terminal Met residues in the peptides with 0–2 Pro residues is controlled by the activated formation of Met(S∴S)^+^ whereas in the peptides with 3–4 Pro residues, by the relative diffusion of the MetS^●+^ and the unoxidized sulfur atom. A decrease in the yields of Met(S∴S)^+^ species with an increase in the number of Pro residues occurs at the expense of an increase in the yields of intramolecular Met(S∴O)^+^ radical cations, and α-(alkylthio)alkyl radicals (α-S) [100]. These findings were similar to those obtained for the photo-induced oxidation of Met-(Pro)_n_-L-Met peptides by the triplet state of CB (see Section 3.2.2, Scheme 12).

### 3.2. Photo-Induced Oxidation

#### 3.2.1. Methionine as the N/C-Terminal and the Internal Amino Acid Residue

The quenching of CB triplets by methionine-containing peptides occurs with rate constants of the order of 2–3 × 10^9^ M^−1^s^−1^ (Table 2). Small differences in the quenching rate constants (indicating the existence of multiple sulfur targets in the quenching process) were interpreted and discussed in reference [16]. The formation of the electron transfer products such as CB^∙−^ radical anion, CBH^∙^ ketyl radical and (S∴N)^+^ and (S∴S)^+^ transients observed in the nanosecond laser flash photolysis indicated that the quenching is electron transfer in nature. As in the case of methionine derivatives, the mechanism of the primary steps in the photooxidation can be described by three primary reactions of the CT complex [CB^∙−^…>S^∙+^]: (i) charge separation (k_sep_) yielding CB^∙−^ and >S^∙+^ radical ions, (ii) proton transfer within the CT complex (k_H_) yielding a ketyl radical CBH^∙^ and an α-(alkylthio)alkyl radical (αS), and (iii) back electron transfer (k_bt_) (Scheme 8). In the case of peptides with an N-terminal methionine residue at low pH, an additional, fourth primary reaction (namely proton transfer from the protonated amino group(-NH_3_^+^) to the CB^∙−^ radical anion within the CT complex (k_NH_ reaction–see Scheme 8) was present. The quantum yields of primary intermediates in the CB-sensitized photooxidation of Met peptides are summarized in Table 7.

An analysis of these results led to conclusions similar to those for methionine derivatives. For peptides with the N-terminal methionine residue (Met-Gly, Met-Gly-Gly, Met-Lys, Met-Met) at low pH, the k_NH_ reaction was responsible for the large values of the quantum yields of CBH^•^ and the (S∴N)^+^ intermediate. At high pH, the charge separation reaction (k_sep_) was the main primary reaction of the CT complex [CB^•−^…>S^•+^] and large values of Φ_CB•−_ and Φ_(S__∴N)+_ were observed. Secondary reactions of the (S∴N)^+^ radical cation in alkaline solutions for Met-Gly peptide (pH 9–11) studied by laser flash photolysis were described in detail in reference [17]. In the case of peptides with C-terminal methionine regardless of pH, the intermolecular dimeric sulfur radical cations (S∴S)^+^ were the main intermediates. The decarboxylation reaction was not observed (or could be neglected in comparison to the other efficient reactions) for Met-containing peptides. This indicates that for peptides with a C-terminal methionine the main irreversible reaction of the sulfur-centered radical cation >S^•+^ was deprotonation leading to the α-(alkylthio)alkyl radical (αS) but a competing decarboxylation reaction could be neglected. This interpretation was additionally confirmed in the steady-state irradiation experiments, where radical coupling stable products CBH-αS were detected [20]. 

In the case of methionine as the internal amino acid residue (Gly-Met-Gly), the results from the CB-sensitized photooxidation were discussed based on the general mechanism presented in Scheme 8. Four primary reactions of CT complex [CB^∙−^…>S^∙+^] could occur: 

(i) charge separation (k_sep_) yielding CB^∙−^ and >S^∙+^ radical ions, (ii) proton transfer within the CT complex (k_H_) yielding a ketyl radical CBH^∙^ and an α-(alkylthio)alkyl radical (αS), (iii) back electron transfer (k_bt_), and (iv) proton transfer from the protonated amino group(-NH_3_^+^) of Gly to the CB^∙−^ radical anion within the CT complex (k_NH_). At low pH the k_NH_ and k_H_ reactions were the main reaction channels (Φ_CBH__∙_ = 0.24) in comparison with the less efficient reaction of charge separation (Φ_CB•−_ = 0.05) (Table 5). It is noteworthy that in this case, the k_NH_ reaction does not lead to the (S∴N)^+^ transient as for an N-terminal methionine residue in peptides. (For expected products from a decay of >S^∙+^ radical ions containing unprotonated amino group in the Gly residue see Scheme 11). In basic solutions, the charge separation reaction (k_sep_) was the main reaction channel yielding CB^∙−^ and >S^∙+^ radical ions (Φ_CB•−_ = 0.21). (S∴N)^+^ cyclic radical cations involving a nitrogen atom from a peptide bond was not observed.

The mechanism of CB-sensitized oxidation of Met-Gly and Gly-Met peptides was also studied by the time-resolved CIDNP technique by Morozova et al. [24] at various pH values. It was shown that at low pH for both peptides a sulfur-centered radical cation >S^•+^ was formed. For Met-Gly, the >S^•+^ radical cation was converted (after deprotonation) to the (S∴N)+ radical cation with five-membered cyclic structure. In basic solutions the main transients for Met-Gly were (S∴N)^+^ radical cations and aminyl type radicals; however, for Gly-Met, the >S^•+^ radical cations and aminyl type radicals were the main transients. 

The mechanism proposed on the basis of the CIDNP experiments correlates with that obtained on the basis of laser flash photolysis experiments with an exception of aminyl radicals. (These radicals were not identified in the laser flash photolysis experiments, see Section 2.2.1).

#### 3.2.2. Linear Peptides with Two Methionine Residues

In the case of Met-Met peptides (Table 8), as was presented in reference [63], the CB-sensitized photooxidation led to formation of intramolecular two-centered three-electron bonded (S∴S)^+^ radical cations that can compete with the formation of intramolecular (S∴N)^+^ radical cations at low pH via the *k*_NH_ reaction (see Scheme 6) and at high pH via cyclization of >S^•+^ radical cations. 

At high pH, the charge separation reaction (*k*_sep_) yielding CB^∙−^ and >S^∙+^ radical ions was shown to be the main primary process involved in the decay of the [CB^∙−^…>S^∙+^] complex and the quantum yield of CB^∙−^ was equal to the sum of quantum yields of the sulfur radical cations (S∴N)^+^ and (S∴S)^+^. At low pH, in addition to the charge separation reaction (*k*_sep_) reaction the proton transfer rection from the protonated amino group(-NH_3_^+^) to the CB^∙−^ radical anion within the CT complex (*k*_NH_ reaction–see Scheme 8) was present. Small differences in the values of quantum yields for (S∴S)^+^ and (S∴N)^+^ radical cations were found for mixed L,D or D,L stereoisomers of Met-Met and L,L or D-D stereoisomers. However, for mixed stereoisomers the quantum yields of (S∴N)^+^ radical cations went down at the expense of higher values for (S∴S)^+^ radical cations in both acidic and basic solutions. In addition, for mixed stereoisomers the quantum yields of (S∴S)^+^ radical cations were more than twice higher of those for L,L and D,D stereoisomers. These observations were rationalized by the higher propensity of ((S∴S)^+^ formation for mixed stereoisomers. 

As was mentioned previously (see Section 3.1.3), the L-Met-(Pro)_n_-L-Met peptides with Met residues located on N- and C- termini were shown to be excellent models of oligopeptides for studying the intramolecular interaction between two sulfur atoms in oligopeptides since this series of Met-(Pro)_n_-Met peptides provides the investigator with the possibility for controlling the S…S distance (Scheme 12). 

This is important because amino acid chains can serve as relay stations for electron transfer processes. As shown in Scheme 8, in the CB-sensitized photooxidation of methionine containing compounds (photochemical path), the sulfur-centered monomeric radical cations (>S^∙+^) were formed. The >S^∙+^ radical cations (regardless of location on N or C termini) can be stabilized by formation of the intramolecular dimeric sulfur radical cations (S∴S)^+^, and (S∴O)^+^ or (S∴N)^+^ intermediates or decay by deprotonation leading to α-(alkylthio)alkyl radicals (αS). The nanosecond laser flash photolysis experiments and the resolution of transient absorption spectra allowed the investigators to monitor kinetics and quantum yields of the intermediates at various time delays after the excitation laser pulse [68]. It was found that a decrease in the quantum yield (Φ) of the (S∴S)^+^ intermediate with the number of proline residues occurred at the expense of quantum yields (Φ) of the other intermediates: (S∴O)^+^/(S∴N)^+^ and αS radicals. However, the dependence of Φ(S∴S)^+^ on the average distance between sulfur atoms was not linear (the largest change in Φ(S∴S)^+^ was observed when the number of proline residues was changed from two to three). These observations were reproduced by Langevin dynamics and statistical mechanical theory showing that for peptides with zero to two Pro residues contact between sulfur atoms was controlled by the activated formation of (S∴S)^+^ but for the peptides with three and four proline residues was controlled by relative diffusion of the >S^∙+^ radical cation and an unoxidized S atom [68]. These findings were similar to those obtained for the radiation-induced oxidation of Met-(Pro)_n_-L-Met peptides by ^●^OH radicals (see Section 3.1.3). 

## 4. Methionine in Cyclic Peptides

**Figure 4:**
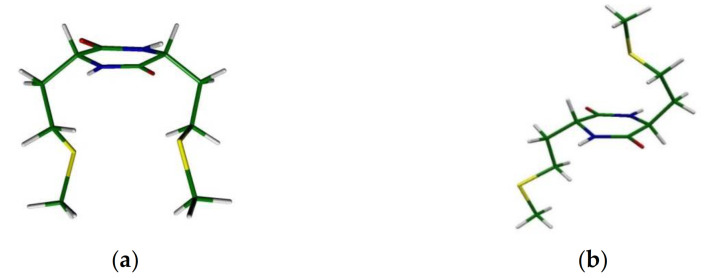
Schematic drawing of structures of (**a**) the cyclic L-Met-L-Met dipeptide and (**b**) the cyclic L-Met-D-Met dipeptide.

Non-extended conformers dominate in the cyclic structures of Met-Met dipeptides due to the expressed tendency toward cis amides. Therefore, the side chains of Met residues in L, L-configured cyclic dipeptides are on the same side of a diketopiperazine ring and, as a consequence, close contacts between them are enhanced (Figure 4a). On the other hand, the side chains of Met residues in L,D-configured cyclic dipeptides are on opposite sides of a diketopiperazine ring and their close contacts are difficult if not impossible, due to the strong steric constraints in the diketopiperazine ring (Figure 4b). Moreover, the presence of free terminal amino and carboxyl groups is eliminated and, thus, stabilization of monomeric sulfur radical cations (MetS^●+^) can come solely from interactions with lone electron pairs on the sulfur atoms in the side chains and/or with lone pairs on the heteroatoms (N- and O-atoms) associated with the peptide bonds.

### 4.1. Radiation-Induced Oxidation

#### 4.1.1. Cyclic Dipeptides with Single Met Residue

Oxidation of c-(Gly-L-Met) dipeptide by ^●^OH radicals was mainly aimed at determining whether in the case of c-(D-Met-L-Met) intramolecular Met(S∴S)^+^ radical cations or intermolecular dimeric radical cations (MetS∴SMet)^+^ are formed (see Section 4.1.2) [13]. The oxidation pattern observed in c-(Gly-L-Met) dipeptide in which there is no chance of forming an intramolecular Met(S∴S)^+^ radical cation is exactly the same as observed in c-(D-Met-L-Met). Similarities in the spectral and kinetic features observed in c-(Gly-L-Met) and c-(D-Met-L-Met) clearly indicate that the mechanisms of OH-induced oxidation of both peptides and the nature of the intermediates are the same for both dipeptides (see Section 4.1.2, Scheme 14). 

#### 4.1.2. Cyclic Dipeptides with Two Met Residues

**Scheme 13:**
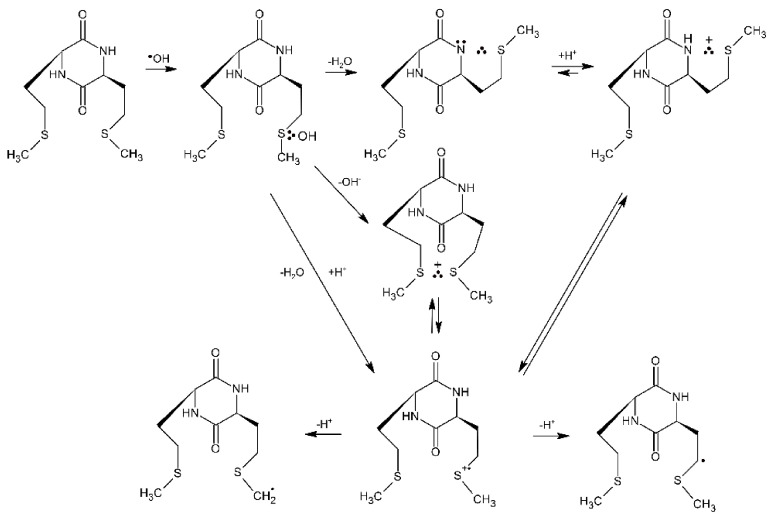
General reaction scheme for ^●^OH-induced oxidation of methionine in c-(L-Met-L-Met).

For the above conformational reasons, cyclic Met-Met dipeptides are suitable models compounds to study reaction of MetS∴OH and MetS^●+^ in oligopeptides and proteins containing multiple and adjacent Met residues (e.g., calmodulin CaM-Ca_4_ (see Section 5.1), human prion proteins hPrP [101], α-synuclein [102]). A small model cyclic dipeptide c-(L-Met-L-Met) (see Figure 3a) was oxidized by ^●^OH radicals generated by pulse radiolysis and the ensuing reactive intermediates were monitored by time-resolved UV-vis spectroscopy and conductometry [12]. Nearly similar radiation chemical yields of intramolecular Met(S∴N) radicals and intramolecular Met(S∴S)^+^ radical cations are formed (see Table 9) in the competitive processes from the primary formed MetS∴OH adduct (Scheme 13).

One feature that requires a note is that the neutral Met(S∴N) radical seems to arise directly from an intermediary MetS∴OH adduct rather than going through the monomeric MetS^●+^ radical cation. Mechanistically, this involves a concerted deprotonation and HO^−^ elimination from MetS∴OH. which frees up an electron pair on the N-atom of the peptide bond. Ultimately, the Met(S∴N) radicals decayed via two-different pH-dependent reaction pathways: (i) at low pH by conversion into additional Met(S∴S)^+^ radical cations via the Met(S∴NH)^+^ intermediate with the rate constant *k*_Met(S__∴N) + H_^+^ = (2.0 ± 0.1) × 10^9^ M^−1^s^−1^, and (ii) at pH close to neutral by a series of consecutive reactions involving, protonation, hydrolysis, and electron transfer followed by decarboxylation of the N-centered radical on Met (not shown in Scheme 13) [12]. The observed decrease of formation of Met(S∴S)^+^ radical cations via pathway (i) with pH is clearly corroborated by the respective *G*(Met(S∴S)^+^ values recorded at various pH values before and after decay of Met(S∴N) (Table 9). Interestingly, an absorption band assigned to Met(S∴S)^+^ in c-(L-Met-L-Met) is characterized by λ_max_ = 520 nm which is red shifted in comparison to λ_max_ = 480 nm of the absorption band of intermolecular dimeric radical cations (MetS∴SMet)^+^ in Met [7] or λ_max_ = 490 nm of the absorption band of intramolecular Met(S∴S)^+^ radical cations in linear L-Met-L-Met dipeptides [9]. This phenomenon can be rationalized by the less favorable overlap of the p orbitals of sulfur atoms in c-(L-Met-L-Met) due to stronger steric constraints of diketopiperazine ring [98,99]. 

**Table 9:** Radiation chemical yields of intramolecular Met(S∴N) and Met(S∴S)^+^ formed during ^●^OH-induced oxidation of the cyclic L-Met-L-Met dipeptide in N_2_O-saturated aqueous solutions.

Type of the Radical	pH 4	pH 4.3	pH 4.9	pH 5.3
Intramolecular Met(S∴N)	0.26 ^(a)^	0.28 ^(a)^	0.25 ^(a)^	0.27 ^(a)^
	0	0	0	0
Intramolecular Met(S∴S)^+^	0.22 ^(a)^	0.23 ^(a)^	0.22 ^(a)^	0.23 ^(a)^
	0.45 ^(a)^	0.45 ^(a)^	0.32 ^(a)^	0.34 ^(a)^

^(a)^ in μM J^−1^; [L-Met-L-Met] = 0.2 mM.

Oxidation of the cyclic dipeptide c-(D-Met-L-Met) by ^●^OH radicals induced by pulse radiolysis was also studied with combined time-resolved UV-vis spectroscopy and conductometry. This isomer has geometric restrictions which should impose limitations on stabilization of intramolecular Met(S∴S)^+^ radical cations (see Figure 3b). In contrast to the previously observed intramolecular stabilization of MetS^●+^ by the unoxidized sulfur atom in the neighboring Met, in the isomer c-(L-Met-L-Met) (Scheme 13 and Table 9), no similar intramolecular stabilization of MetS^●+^ was observed in c-(D-Met-L-Met). One feature that requires a note at this point is that the species absorbing in the 480–490 nm range has to be assigned to intermolecular dimeric radical cations (MetS∴SMet)^+^ based on the spectral and kinetic features observed in c-(Gly-L-Met) (see Section 4.1.1). 

**Scheme 14:**
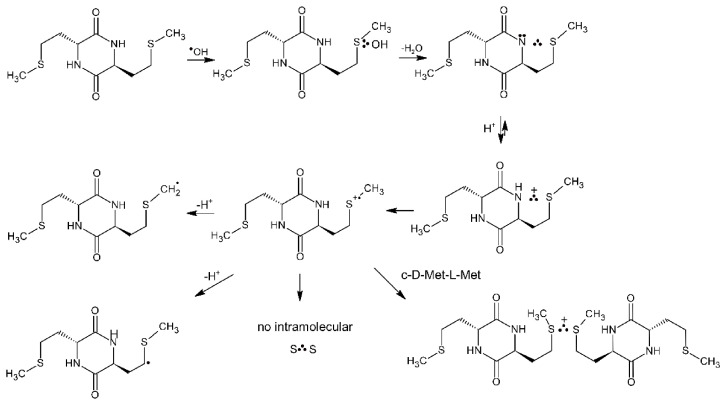
General reaction scheme for ^●^OH-induced oxidation of methionine in c-(D-Met-L-Met).

However, formation of Met(S∴N) occurs via an analogous concerted deprotonation and HO^−^ elimination mechanism from MetS∴OH as in c-(L-Met-L-Met) (Scheme 14). 

### 4.2. Photo-Induced Oxidation

#### 4.2.1. Cyclic Dipeptides with Single Met Residue

**Figure 5:**
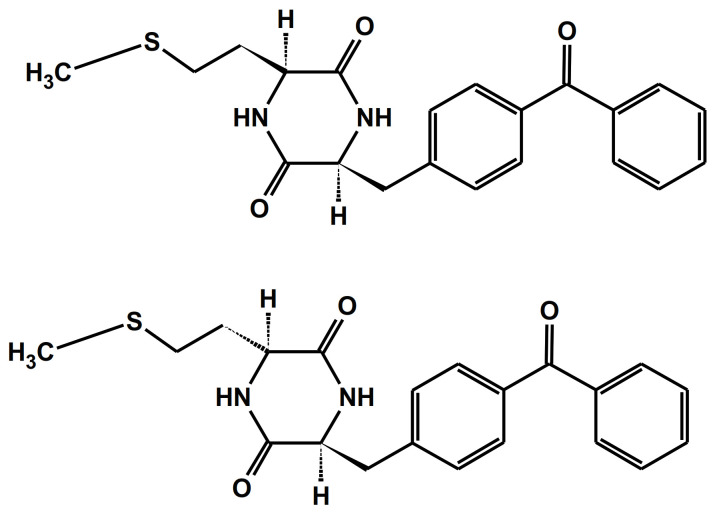
Structures of diketopiperazine-based benzophenone-methionine dyads: c-(L-BP-L-Met) (top) and c-(L-BP-D-Met) (bottom).

One of the simplest model compounds for sensitized photooxidation are diketopiperazine-based benzophenone-methionine dyads: c-(L-BP-L-Met) and c-(L-BP-D-Met) with their structures presented in Figure 5 [76]. They allowed the investigators to study the intramolecular quenching of BP triplets by Met in conformationally controlled donor-acceptor pairs and to study the dynamics of the intermediates formed in the oxidation process.

The combined results from time-resolved and steady-state experiments showed that for c-(L-BP-L-Met), the quenching occurred via electron transfer from the sulfur atom to the benzophenone triplet (via the [BP^∙−^…>S^∙+^] complex) was followed by the proton transfer reaction that led to a biradical intermediate with the quantum yield Φ_ketyl_ = 0.6. These biradicals recombined to form a cyclic stable product via C-C coupling reaction. For the c-(L-BP-D-Met) dyad, with its two reactive moieties on the opposite side of diketopiperazine ring, the quenching process and formation of biradical were shown to be less effective (Φ_ketyl_ < 0.18). It was shown that differences in the reaction rates for both isomeric BP-Met dyads were attributed to the differences between the distribution of the carbonyl/sulfur atom distances in the two stereoisomers.

#### 4.2.2. Cyclic Dipeptides with Two Met Residue

Cyclic Met-Met dipeptides are also suitable model compounds for proteins to study the photo-induced oxidation. They have the unique features of having no terminal groups and therefore they are good model to study interactions between a side chain and peptide bonds. 

The mechanism of the CB-sensitized photooxidation of c-(L-Met-L-Met) with Met residues in the cis position was studied using nanosecond flash photolysis techniques [63]. The photochemical path of c-(L-Met-L-Met) oxidation led to the >S^∙+^ radical cation (as presented in Scheme 8). This radical cation can decay in two competing reactions: the formation of intramolecular dimeric sulfur radical cations (S∴S)^+^ and a deprotonation yielding αS radicals. Similar to the results presented above for the radiation-induced oxidation, the efficiency of intramolecular (S∴S) ^+^ formation was relatively large (Φ(_S__∴S)+_ = 0.14 (see Table 8) showing that this reaction was a main path of >S^∙+^ radical cation decay. The mechanism of (S∴S) ^+^ formation was shown to be similar to that presented in Scheme 13 for the radiation-induced oxidation of c-(L-Met-L-Met).

The CB-sensitized photooxidation of cyclic dipeptides containing methionine residues was also studied using time-resolved CIDNP [25]. For c-(D-Met-L-Met) with Met residues on the opposite side of the ring and for c-(Gly-Met) the sulfur-centered radical cations >S^∙+^ were detected. In the case of c-(L-Met-L-Met the intramolecular cyclic (S∴S)^+^ transients with three-electron bond between two sulfur atoms were detected.

## 5. Methionine in Proteins

### 5.1. Radiation-Induced Oxidation

Pulse radiolysis is a very useful method for studies of free radical processes in biological systems, including proteins. However, the vast majority of studies was focused on the generation of unstable intermediates in proteins through one-electron reduction [103]. There were also some attempts aimed at studying the oxidation processes in proteins. However, they only concerned the oxidation of amino acids located in proteins which are very prone to oxidation, such as Trp or Tyr [5,104,105,106,107]. To the best of our knowledge, only two papers addressed radiation-induced oxidation of Met in proteins and the subsequent steps following oxidation [108,109]. 

**Scheme 15:**
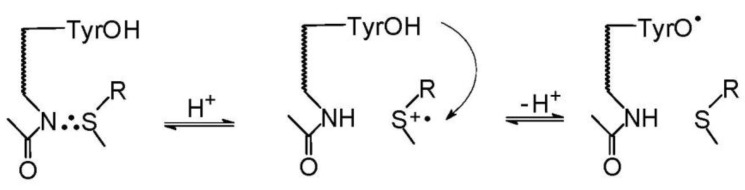
electron transfer between Met(S∴N)^+^ and Tyr in CaM-Ca_4_ (adapted from [109]).

Thioredoxin Ch1 (Trx) from Chlamydomonas reinhardtii contains two Met residues: Met^41^ and Met^79^. The one-electron oxidation of the W35A mutant of Trx by pulse radiolytically generated N_3_^●^ radicals led to a transient absorption spectrum with three absorption bands characterized by λ_max_ = 390, 420, and 480 nm. The absorption band with λ_max_ = 420 nm indicated formation of TyrO^●^ radicals via direct reaction of Tyr with N_3_^●^ radicals. On the other hand, the absorption bands with λ_max_ = 390 and 480 nm were assigned to Met(S∴O)^+^ which results from the interaction of the monomeric sulfur radical cation (MetS^●+^) with the O-atom of the carbonyl function of Phe^31^ (which is less than 4 Å away from S-atom) and Met(S∴S)^+^ which results from the interaction of MetS^●+^ with the S-atom of Cys^49^, respectively. With time evolution, no oxidized products derived from Met were detected but there was a further increase of the absorption band assigned to TyrO^●^ radicals. An intramolecular electron transfer from the Tyr residue to Met-derived radicals seems to be responsible for the latter observation [108] (see Scheme 15 for an analogous reaction involving Met(S∴N)^+^ radical cation).

In turn, calmodulin (CaM-Ca_4_) (a regulatory “Ca-sensor protein”) contains nine Met residues: Met^36^, Met^51^, Met^71^, Met^72^, Met^76^, Met^109^, Met^124^, Met^144^, and Met^145^ which are located in a variety of local environments. Therefore, the stabilization of the monomeric sulfur radical cation (MetS^●+^) can be realized in various ways depending upon local structures. The one-electron oxidation of CaM-Ca_4_ using pulse radiolytically generated ^●^OH radicals led to a transient absorption with λ_max_ = 390 nm which shows a close similarity to that characteristic for S∴N-bonded radical cations observed in cyclic Met-Met dipeptides [12,13]. The lack of the characteristic spectrum of intramolecular S∴S-bonded radical cation, despite the proximity of methionine residues in two pairs Met^71^/Met^72^ and Met^144^/Met^145^ can be rationalized by local steric constraints which prevent close contact of their side chains [13]. With time evolution, the transient absorption is shifted to λ_max_ = 410 nm which is characteristic for TyrO^●^ radicals. This observation was rationalized in terms of intramolecular electron transfer from Tyr to Met(S∴N)^+^ radical cations (see Scheme 15) [109].

### 5.2. Photo-Induced Oxidation 

Despite the significant role of protein oxidation reactions in biology and medicine the sensitized photooxidation of Met within proteins was the subject of only a few papers. This may be due to complexity of the reactants, the diversity of possible target positions in the oxidation of proteins, and the experimental difficulties in analyzing the oxidation products. The mechanism of CB-sensitized photooxidation of plant cytokinin-specific binding proteins (VrPhBP) was recently studied using nanosecond flash photolysis and various methods for analyzing the stable photoproducts (chemical analysis, silver-staining gel electrophoresis, chromatography, and mass spectrometry including peptide mapping by proteolysis and coupling with chromatography) [110]. The results indicated oxidation of methionine and tyrosine residues within the protein.

**Scheme 16:**
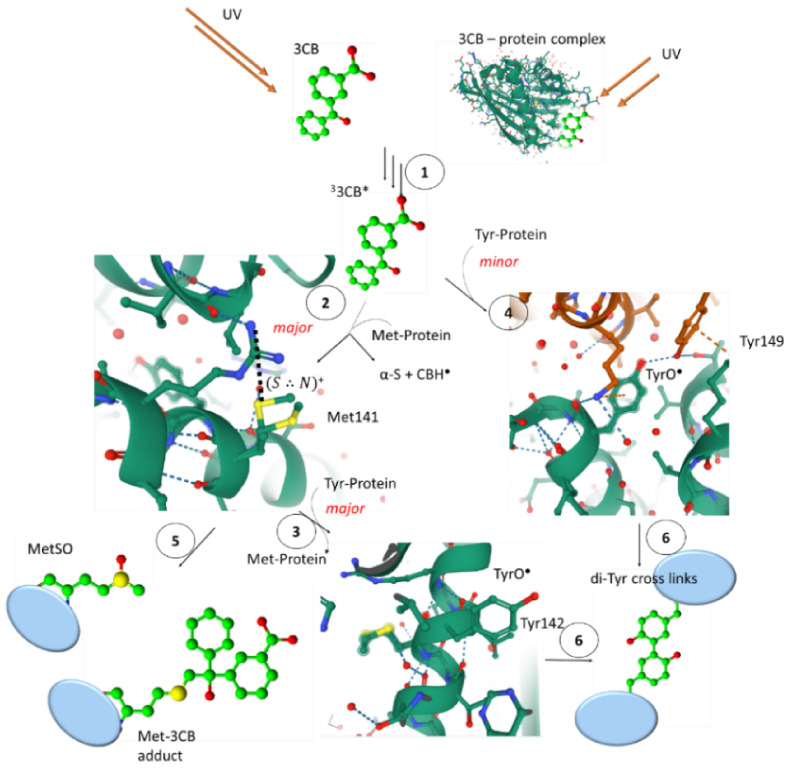
Graphical scheme reaction of the mechanism of VrPhBp protein oxidation sensitized by CB in aqueous solutions methionine in Gly-Met-Gly tripeptide (adapted from [110]).

Based on the crystallographic structure of the VrPhBP protein and taking into account knowledge from similar studies for model amino acids and peptides, methionine (Met^141^) and tyrosine (Tyr^142^) residues were suggested as being the most prone to oxidation. It was shown that the CB triplets were mainly quenched by methionine and partially by tyrosine residues via the electron transfer mechanism leading to the formation of sulfur-centered monomeric radical cations >S^∙+^ (stabilized by (S∴N)^+^ formation), tyrosyl radicals TyrO^∙^ and ketyl radicals CBH^∙^. As was already shown for the model peptide (Met-enkephalin) [61], the intramolecular electron transfer from the Tyr residue to the oxidized sufur atom of the Met residue can take place leading to regeneration of the Met residue and formation of additional TyrO^∙^ radicals. The presence of the transient species observed in the time-resolved experiments were additionally confirmed by the detection of such stable products as methionine sulfoxide, the Met-CBH adduct (radical coupling products (αS-CBH) and dityrosine cross links (see Scheme 16). The results from the study of sensitized photooxidation of model compounds including methionine derivatives and peptides were very helpful to understand the mechanism of sensitized photoxidation of proteins containing methionine residues.

## 6. Conclusions

A wealth of knowledge has been accumulated over the years concerning the mechanistic understanding of one-electron oxidation of methionine in various structural environments using two-complementary radiation and photochemical time-resolved techniques such as pulse radiolysis and laser flash photolysis coupled with UV-vis spectrophotometry, conductometry, ESR spectroscopy and CIDNP. Creation of a variety of very reactive transients such as ^●^OH-adducts to methionine (MetS∴OH), monomeric sulfur radical cations (MetS^●+^), intramolecularly (S∴N)-bonded radicals (Met(S∴N)) or radical cations (Met(S∴N)^+^), α-alkylthio)alkyl radicals (αS1, αS2), α-aminoalkyl radicals (αN) intermolecularly S∴S-bonded dimeric radical cations ((MetS∴SMet)^+^), intramolecularly S∴S-bonded radical cations (Met(S∴S)^+^), and intramolecularly S∴O-bonded radical cations (Met(S∴O)^+^), which are precursors for the subsequent final products responsible for protein damage, requires a knowledge of their spectral parameters as well as the mechanisms of their formation and decay along with appropriate kinetic parameters. Neighboring group participation seems to be an essential parameter which controls one-electron oxidation of methionine by various one-electron oxidants in various structural and pH environments. Provided that photo- and radiation-induced reaction pathways lead to the formation of a common intermediate, namely the monomeric sulfur-centered radical cation (MetS^•+^), its secondary reactions in peptides and proteins will be the same. This review was also aimed at demonstrating the complementarity of photo- and radiation-chemical studies using the example of methionine oxidation processes.

Future studies on photo- and radiation-induced oxidation of Met containing compounds aimed at analysis of stable products formed during the steady state photo- and γ-irradiations (similar to those reported in the references [20,22,59,76] are expected to help in understanding the overall mechanism of oxidation from short-lived intermediates to the final products. 

## Figures and Tables

**Table 3:** Quantum yields of primary (Φ) and secondary (Φ”) intermediates and CO_2_ in the CB-sensitized photooxidation of Met.

	pH	Φ_CBH_^∙^	Φ_CB_^∙−^	Φ_CBH_^∙^ + Φ_CB_^∙−^	Φ_(S__∴N)_^+^	Φ_(S__∴S)_^+^	Φ’’ _CBH_^∙^	Φ_CO2_-	Lit
Methionine	7	0.11	0.27	0.38	0	0.23	0.25	0.28	[18]
Methionine	11	0.05	0.66	0.71	0.38 ^(a)^	0.10	0.66 ^(b)^	0.55	[18,67]

error ±20%; ^(a)^ short-lived transient Φ**_(S__∴N)_^+^,** taken at ~20 ns after the laser pulse; ^(b)^ Φ’’_CB_^−^.

**Table 4:** Quantum yields of primary intermediates and CO_2_ in the CB-sensitized photooxidation of Met derivatives.

Met Derivatives	pH	Φ_CBH_^∙^	Φ_CB_^∙−^	Φ_CBH_^∙^ + Φ_CB_^∙−^	Φ_(S__∴N)_^+^	Φ_(S__∴S)_^+^	Φ_CO2^−^_	Lit
Met-OCH_3_	6	0.39	0	0.39	0.20	0.04	0	[19,75]
Met-OCH_3_	10	0	0.43	0.43	0.28	0.04	0	[19,75]
Met-NH_2_	5	0.39	0	0.39	0.28	0.10	0	[19,75]
Met-NH_2_	10	0	0.48	0.48	0.12	0.07	0	[19]
N-Ac-Met	7	0.12	0.29	0.41	0	0.28	0.05	[18,19]
N-Ac-Met	10	0.13	0.29	0.42	0	0.27	−	[19,75]
N-Ac-Met-OCH_3_	6	0.22	0.04	0.26	<0.05	0.07	0	[19]
N-Ac-Met-NH_2_	6	0.19	0.07	0.26	0	0.09	0	[19]
N-Ac-Met-NHCH_3_	7	0.32	<0.02	0.34	0	0	0	[22]

errors ± (10–20)%.

**Table 5:** The radiation chemical yield (*G*) ^(a)^ of radicals and radical cations and their percentage contribution (in parenthesis) to the total yield of radicals formed in the reaction of ^●^OH radicals with Gly-Met-Gly ^(b)^ [74].

Time	α-N	α-S	α-C	Met(S∴N)^+^	Met(S∴S)^+^
1.4 μs	0.34 (57.1%)	0.14 (23.6%)	0.052 (8.6%)	0.035 (5.9%)	0.029 (4.8%)
3.5 μs	0.35 (58.7%)	0.13 (21.8%)	0.061 (10.2%)	0.029 (4.8%)	0.027 (4.5%)

^(a)^ G-values are in μmol J^−1^ units; ^(b)^ [Gly-Met-Gly] = 0.2 mM.

**Table 6:** The radiation chemical yield (G) ^(a)^ of radicals and radical cations and their percentage contribution (in parenthesis) to the total yield of radicals formed in the reaction of ^●^OH radicals with N-Ac -Gly-Met-Gly ^(b)^ [74].

Time	MetS∴OH	α-S	α-C	Met(S∴N)^+^	Met(S∴S)^+^
1.4 μs	0.18 (30.6%)	0.17 (29.9%)	0.05 (8.1%)	0.10 (17.7%)	0.08 (13.6%)
3.5 μs	0.07 (11.8%)	0.29 (49.0%)	0.02 (3.8%)	0.11 (19.2%)	0.10 (16.3%)

^(a)^ G-values are in μmol J^−1^ units; ^(b)^ [N-Ac-Gly- Met-Gly] = 0.2 mM.

**Table 7:** Quantum yields of primary intermediates and CO_2_ in the CB-sensitized photooxidation of Met-containing linear peptides.

Met-Containing Peptides ^(a)^	pH	Φ_CBH_^∙ (b)^	Φ_CB_^∙− (b)^	Φ_CBH_^∙^ + Φ_CB_^∙− (b)^	Φ_(S__∴N)_^+ (b)^	Φ_(S__∴S)_^+ (b)^	Φ_CO2_- ^(c)^	Lit
Met-Gly	6	0.25	0.13	0.38	0.18	~0.04	0.01	[16,18]
Met-Gly	11	0.06	0.55	0.61	0.51	~0.04	0.01	[16,18,75]
Gly-Met	6	0.15	0.22	0.37	0	0.22	0.02	[18]
Gly-Met	11	0.10	0.32	0.42	0	0.24	0.02	[18]
Met-Gly-Gly	6	~0.29	~0.06	~0.35	0.14	~0.08	0.01	[16]
Met-Gly-Gly	11	0.09	0.49	0.58	0.30	<0.03	−	[16]
Gly-Gly-Met	6	0.19	0.25	0.44	0	0.24	0.02	[16]
Gly-Gly-Met	11	0.13	0.30	0.43	0	0.29	−	[16]
Gly-Met-Gly	6	0.24	0.05	0.29	0	0.08	0.02	[16]
Gly-Met-Gly	11	0.10	0.21	0.31	0	0.07	−	[16]
Met-Lys	6	0.55	~0.03	0.58	0.33	0	0	[20]
Lys-Met	6	0.63	0	0.63	0	0.19	0	[20]
Met-Enkephalin	7	−	−	0.82	0	0	0	[61]

^(a)^ [CB] = 2–4 mM, [Met peptides] = 5–20 mM; ^(b)^ error ± 20%; ^(c)^ error ± 50%.

**Table 8:** Quantum yields of primary intermediates and CO_2_ in the CB-sensitized photooxidation of peptides containing two methionine residues.

Met-Containing Peptides ^(a)^	pH	Φ_CBH_^∙ (b)^	Φ_CB_^∙− (b)^	Φ_CBH_^∙^ + Φ_CB_^∙− (b)^	Φ_(S__∴N)_^+ (b)^	Φ_(S__∴S)_^+ (b)^	Φ_CO2_- ^(c)^	Lit
L-Met-L-Met	6	0.23	0.15	0.38	0.19	0.07 ^(d)^	<0.01	[63]
L-Met-L-Met	10	0.13	0.41	0.54	0.43	0.01 ^(d)^	<0.01	[63]
D-Met-D-Met	6	0.24	0.18	0.42	0.27	0.06 ^(d)^	-	[63]
D-Met-D-Met	10	0.09	0.40	0.49	0.41	~0.02 ^(d)^	-	[63]
L-Met-D-Met	6	0.17	0.23	0.40	0.18	0.15 ^(d)^	<0.01	[63]
L-Met-D-Met	10	0.08	0.37	0.45	0.34	0.06 ^(d)^	<0.01	[63]
D-Met-L-Met	6	0.15	0.25	0.40	0.15	0.16 ^(d)^	<0.01	[63]
D-Met-L-Met	10	0.10	0.38	0.48	0.36	0.06 ^(d)^	<0.01	[63]
c-(L-Met-L-Met)	7	0.17	0.19	0.36	0	0.14 ^(d)^	-	[63]

^(a)^ [CB] = 2 mM, [Met-peptides] = 0.5 mM; ^(b)^ errors ± 10–20%; ^(c)^ error ± 50%; ^(d)^ intramolecular (S∴S)^+^ (adapted after [3]).

## Data Availability

Not applicable.
